# Early bioenergetic evolution

**DOI:** 10.1098/rstb.2013.0088

**Published:** 2013-07-19

**Authors:** Filipa L. Sousa, Thorsten Thiergart, Giddy Landan, Shijulal Nelson-Sathi, Inês A. C. Pereira, John F. Allen, Nick Lane, William F. Martin

**Affiliations:** 1 Institute of Molecular Evolution, University of Düsseldorf, 40225 Düsseldorf, Germany; 2 Institute of Genomic Microbiology, University of Düsseldorf, 40225 Düsseldorf, Germany; 3 Instituto de Tecnologia Química e Biológica, Universidade Nova de Lisboa, Oeiras, Portugal; 4 School of Biological and Chemical Sciences, Queen Mary, University of London, London, UK; 5 Research Department of Genetics, Evolution and Environment, University College London, Gower Street, London, UK

**Keywords:** transition metals, acetogens, methanogens, sulfate reducers, origin of life, hydrothermal vents

## Abstract

Life is the harnessing of chemical energy in such a way that the energy-harnessing device makes a copy of itself. This paper outlines an energetically feasible path from a particular inorganic setting for the origin of life to the first free-living cells. The sources of energy available to early organic synthesis, early evolving systems and early cells stand in the foreground, as do the possible mechanisms of their conversion into harnessable chemical energy for synthetic reactions. With regard to the possible temporal sequence of events, we focus on: (i) alkaline hydrothermal vents as the far-from-equilibrium setting, (ii) the Wood–Ljungdahl (acetyl-CoA) pathway as the route that could have underpinned carbon assimilation for these processes, (iii) biochemical divergence, within the naturally formed inorganic compartments at a hydrothermal mound, of geochemically confined replicating entities with a complexity below that of free-living prokaryotes, and (iv) acetogenesis and methanogenesis as the ancestral forms of carbon and energy metabolism in the first free-living ancestors of the eubacteria and archaebacteria, respectively. In terms of the main evolutionary transitions in early bioenergetic evolution, we focus on: (i) thioester-dependent substrate-level phosphorylations, (ii) harnessing of naturally existing proton gradients at the vent–ocean interface via the ATP synthase, (iii) harnessing of Na^+^ gradients generated by H^+^/Na^+^ antiporters, (iv) flavin-based bifurcation-dependent gradient generation, and finally (v) quinone-based (and Q-cycle-dependent) proton gradient generation. Of those five transitions, the first four are posited to have taken place at the vent. Ultimately, all of these bioenergetic processes depend, even today, upon CO_2_ reduction with low-potential ferredoxin (Fd), generated either chemosynthetically or photosynthetically, suggesting a reaction of the type ‘reduced iron → reduced carbon’ at the beginning of bioenergetic evolution.

## 1. Introduction

Life is a net exergonic chemical reaction, it releases energy to go forward. Many settings have been proposed as the site for the chemical synthesis for life's building blocks [1] and the ignition of the continuous chemical reaction that includes us as its descendants. Such proposed settings include oceans or ponds of organic soup stocked either by ultraviolet light-dependent organic synthesis [2] or by organics delivered from space [3]; borate evaporites [4]; terrestrial zinc-rich hydrothermal settings [5]; ice [6] or pumice [7]—to name but a few. However, since their discovery, submarine hydrothermal vents stand out among the possible environments for life's origin, holding particular promise for understanding the transition from geochemistry to biochemistry [8–12]. Submarine hydrothermal vents harbour highly reactive chemical environments with far-from-equilibrium conditions, being rich in gradients of redox, pH and temperature. Furthermore, hydrothermal vents generate spontaneously, forming systems of inorganic microcompartments [9,13] that, in an origin-of-life context, could readily serve to concentrate the chemical reaction products and substrates that form there, at their site of their synthesis. The far-from-equilibrium condition is important because it harbours the potential for exergonic chemical reactions [14]. Concentration is important as a prerequisite for autocatalysis and for self-regulation, defining characteristics of living systems [15].

## 2. Alkaline hydrothermal vents

A number of submarine hydrothermal vents have been studied. They differ in the specific temperature and the chemical compositions of their effluent fluid [10,16]. Most are located at the seafloor spreading zone, that is, directly above magma chambers. As such, their effluent comes directly into contact with magma and emerges at the vent–ocean interface with a temperature often exceeding 300°C. Such ‘black smokers’ are not only very hot, but they are also short-lived, lasting in the order of decades. In contrast, off-ridge vents of the kind exemplified by Lost City [13,17] are situated several kilometres away from the spreading zone; hence the water circulating through them makes no contact with magma and emerges at a temperature of around 70–90°C. Unlike vent systems that reside directly on the spreading zone, off-ridge vents can be stably active over geological time; Lost City has been active for about 120 000 years [18]. The chemical disequilibrium at vents arises from the contact between their reducing, H_2_-containing effluent with CO_2_-containing ocean water, which is more oxidized. Hydrothermal vents harbour up to 50 mM H_2_ [10]. At Lost City, the H_2_ concentration of the alkaline effluent (approx. pH 9–10) is about 10 mM [19].

Hydrogen is a reducing agent: an electron donor. Given a suitable acceptor, of which there are many [20], hydrogen is a source of energy. The H_2_ in hydrothermal vents comes from a geochemical process called serpentinization [21–25]. Serpentinization takes place because most of the suboceanic crust consists of iron–magnesium silicates like olivine, in which the redox state of the iron is Fe^2+^. Seawater penetrates the ocean floor crust, reaching depths on the order of 3–5 km below the surface. There at high pressure and temperatures of around 150°C, water oxidizes the Fe^2+^ to Fe^3+^, the water being reduced to H_2_ with the oxygen in water being retained in the rock as iron oxides. Bach *et al.* [21] write the serpentinization reaction as in equation (2.1) whereas Sleep *et al*. [22] represent the main redox reaction as in equation (2.2).

Mg_2.85_Fe_0.15_Si_2_O_5_(OH)_4_ is serpentinite, the mineral from which the process is named, and 4Fe_3_O_4_ is magnetite, the mineral containing the oxidized iron. During serpentinization, a cubic metre of olivine eventually yields about 500 mol of H_2_ [24]. The heated water convects back up to the ocean floor, carrying H_2_ and other reaction products with it. The production of H_2_ via serpentinization has probably been taking place since there was liquid water on the Earth [22], and it continues today because, relative to the amount of water on the Earth, the amount of Fe^2+^ in the crust is inexhaustible. The Mg(OH)_2_ in equation (2.1) produces alkaline hydrothermal fluid in agreement with the pH observed for Lost City effluent [17].

Lost City effluent also contains about 1 mM methane of abiogenic origin [19,26], along with about 100 µM formate of abiogenic origin [27] and traces of longer hydrocarbons up to pentane [19]. This indicates that serpentinization in the crust at Lost City reduces CO_2_ to an organic product spectrum. The magma-hosted black smoker systems are less conducive to organic synthesis in the crust, because, above approximately 250°C, carbon in contact with water becomes CO_2_ [28,29], and magma has a temperature of around 1200°C. If Lost City harbours organic synthesis today, then organic synthesis in similar serpentinizing systems should also have been possible 4 billion years ago. While the composition of ocean water has changed since then [30], the composition of the seafloor crust, where serpentinization reactions take place, has remained roughly constant, as indicated by the zircon compositions of Early (Hadean) mantle-derived melts [31].

The chemical disequilibrium at off-ridge hydrothermal vents on the early Earth was, in all likelihood, much greater than that today. This is because there was much more CO_2_ in the atmosphere, perhaps up to 1000 times more [32,33], meaning that there would have been much higher CO_2_ concentrations in the ocean. Hence when H_2_ in the convective currents of serpentinizing systems made contact with marine CO_2_ (stemming *inter alia* from volcanos) there was disequilibrium and potential for organic synthesis.

How much potential do vents harbour for the synthesis of what kind of organic products? Shock and co-workers have studied the question of organic synthesis at hydrothermal vents from the thermodynamic standpoint, and what they find is encouraging from an origin-of-life perspective (reviewed in [14]). They find that CO_2_ reduction and organic synthesis is thermodynamically favoured—exergonic—when reduced vent fluid mixes with more oxidized ocean water at the vent–ocean interface, and hence can take place under the conditions presented by hydrothermal vents. This is true for the synthesis of carboxylic acids, alcohols and ketones [28,29], amino acids and proteins [34] and total microbial cell mass [35,36].

The nature and proportion of organic products that are thermodynamically most favoured depend upon the specific chemical conditions, for example, H_2_ availability, temperature and the redox state of the environment [14,37,38]. Under the conditions found at Lost City, for example, the overall synthesis of microbial cell mass from H_2_, CO_2_ and NH_3_ is exergonic in the temperature range 50–125°C [36].


2.1
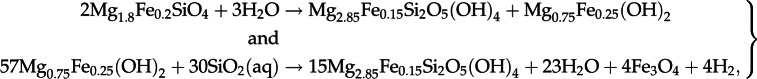



2.2
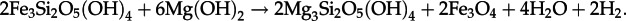


Such findings are broadly consistent with the findings of Thauer *et al.* [39] that under conditions relevant for microbes, in the reaction of H_2_ with CO_2_, the equilibrium lies on the side of reduced carbon compounds, which is why acetogens can grow from the reaction2.3
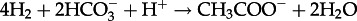
with *ΔG*°*′* = –104.6 kJ mol^−1^ [39] and methanogens can grow from the reaction2.4
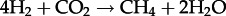
with *ΔG*°*′* = –131 kJ mol^−1^ [40] as their sole source of energy, respectively. They harness (conserve) chemical energy from those reactions to push the life process forward. For a prokaryote, the main energetic cost in the life process is amino acid and protein synthesis, which consumes about 75 per cent of the cell's ATP budget, with RNA monomer and polymer synthesis consuming only about 12 per cent [41].

Because hydrothermal vents present conditions where the synthesis of proteins from H_2_, CO_2_ and NH_3_ would be an exergonic process [14,34], they truly appear to be special among the various settings that have been considered for the origin of life. Hydrothermal vents are particularly rich in chemical and thermodynamic similarities to the core energy releasing reactions of modern acetogens and methanogens [42,43], lineages that over 40 years ago—before the discovery of either hydrothermal vents or archaebacteria—were proposed to be the most ancient microbes, because they are anaerobic chemoautotrophs that live from the H_2_–CO_2_ redox couple [44].

**Figure 1.**
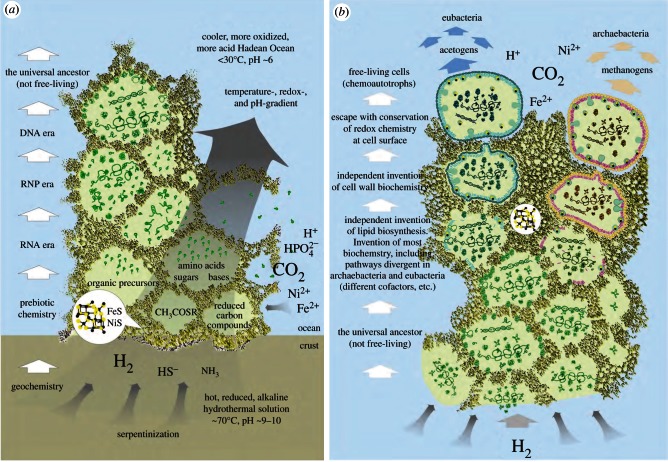
A scheme for the origin of cells [42,46].

The far-from-equilibrium conditions and favourable thermodynamic setting of hydrothermal vents do not indicate which chemical syntheses will occur, merely which are energetically possible [14]. The nature of catalysts present can also influence the kinds of products that are formed [45], depending on whether the reaction is thermodynamically controlled (the most stable products accumulate) or kinetically controlled (the most rapidly synthesized products accumulate). Here, we revisit a model for the origin of life (figure 1) as set forth previously in these pages while incorporating newer findings.

## 3. Getting from rocks and water to cells

One can assume that off-ridge vents such as Lost City were more prevalent on the early Earth than today [11,22,23]. But the chemistry at a Hadean vent–ocean interface would have been slightly different, with abundant Fe^2+^ [30] and far more CO_2_ [32,33] in the ocean. Given that, and given the presence of sufficient sulfide in the hydrothermal fluid to steadily precipitate transition metal sulfides (FeS, NiS and the like) upon mixing of effluent and ocean water at the vent interface [9], the model posits that hydrothermal flux deposited a mound at the vent, consisting crucially of transition metal sulfides, among other possible minerals. The premise that a Hadean mound could have existed is underpinned by the report of fossilized metal sulfide hydrothermal mounds 360 Myr of age—the rocks that gave rise to this model in the first place [47]—found in Tynach, Ireland by Russell and co-workers [48]. These mounds reveal elaborate systems of naturally forming, inorganically walled microcompartments (on the order of 1 µm–1 mm in diameter). The walls of these compartments unite two very important properties for early chemical evolution: catalysis and compartmentation.

The catalysis was provided by the transition metals and transition metal sulfides themselves of the hydrothermal mound. That is, the microcompartments have inorganic walls made of essentially the same catalysts that are commonly found in redox-active enzymes of modern microbes [9,49–51] and commonly used in organometallic catalysis in the chemical industry [52,53]. This is an important similarity to modern anaerobic autotrophs, whose enzymes of core carbon and energy pathways are replete with FeS, FeNiS and MoS (WS) centres, FeS and NiS usually being incorporated into proteins via cysteine residues [54–58], Mo and W usually being complexed by thiols of the molybdopterin cofactor MoCo [59]. Importantly, such FeS and FeNiS centres are particularly common in H_2_-oxidizing and CO_2_-reducing proteins of anaerobic prokaryotes [60]. Because the nature of the catalysts at the interface of gases (H_2_, CO_2_, N_2_, H_2_S, SO_2_) and organic matter that (i) we posit to be present at the mound and (ii) occur in acetogens and methanogens is very similar, some congruence between the kinds of products obtained in the two systems is not an unreasonable proposition [45].

Interfacing with the CO_2_-rich seawater at the vent was highly reduced, H_2_-containing, vent effluent that probably contained some reduced carbon compounds itself, as Lost City does today [19,26,27], stemming from serpentinization reactions deep within the crust. How reducing might the effluent in an ancient serpentinizing system have been? A clue is provided by the mineral awaruite (Ni_3_Fe), an alloy of metallic iron and nickel, Fe^±0^ and Ni^±0^, that is quite commonly found in serpentinizing systems and that is produced from Fe^2+^ and Ni^2+^ minerals via reduction to the raw metals *in situ* by H_2_ [25,61]. Awaruite forms only when sulfide concentrations are very low, but that is not the point, nor is the point that Fe^±0^ readily reduces CO_2_ to acetate and formate under hydrothermal conditions [62]. The point is that in the temperature range 150–300°C, activities of H_2_ exceeding 200 mM are needed for awaruite to form [25]. To stress the point, 200 mM H_2_ represents a very large amount of hydrogen and a very reducing environment. The presence of awaruite in serpentinizing systems thus clearly indicates that at some point during their lifespan, serpentinizing systems regularly generate extremely reducing conditions on the order of greater than or equal to 200 mM H_2_, which would be conducive to organic synthesis, as Klein & Bach [25] point out. So while conditions at a Hadean-serpentinizing system cannot be pinpointed, we can say that serpentinizing systems do, in principle, have the reducing power at hand that would be needed for making organic compounds out of CO_2_ (or even elemental iron and nickel out of their salts).

For the building blocks of life to form, nitrogen is also essential. Where does reduced nitrogen come from? Some modern vent systems contain up to 1 mM NH_3_ [10], although it is not known that serpentinization is responsible for N_2_ reduction. However, simulated hydrothermal conditions of 300°C and high pressure readily convert nitrite into NH_3_ [63], and under very mild conditions, FeS minerals can reduce N_2_ to NH_3_ at yields approaching 0.1 per cent [64], whereby native Fe^±0^ is also an effective catalyst of N_2_ reduction under hydrothermal conditions [65], recalling once again awaruite as an indicator of serpentinization [25]. So it should be safe to assume that reduced nitrogen would have been available in a Hadean-serpentinizing hydrothermal system. Phosphate is more soluble at pH 6 than at pH 8 and was not likely to have been globally abundant in the early oceans [11,30], although there was surely considerable local phosphate input from volcanos [66,67]. Phosphate was possibly concentrated at hydrothermal vents, however, via the mineral brucite—Mg(OH)_2_—driven precipitation processes that Holm *et al.* [68] suggest to have involved sepentinization.

The foregoing would provide a mixture of H_2_, CO_2_ and NH_3_ similar to that assumed by Amend *et al.* [14] for synthesis of chemical constituents of cells, in an environment laden with transition metal catalysts, with abundant hydrothermal HS^–^. That is a starting point for the synthesis of compounds with high-energy bonds. Under mild laboratory conditions, Heinen & Lauwers [69] reduced CO_2_ to a spectrum of sulfur containing organic products, including methylsulfide (CH_3_SH), using FeS as the catalyst, and Huber & Wächtershäuser [70] reacted CH_3_SH and CO under mild conditions (ambient pressure, 100°C), using FeS, NiS or Ni^2+^ as catalysts, obtaining up to 40 mol% of the thioester methylthioacetate (or acetyl methylsufide, CH_3_COSCH_3_). Thioesters are energy-rich compounds because the thioester bond has a large free energy of hydrolysis, *ΔG*°′ = –32 kJ mol^−1^ [71]. Starting from such simple chemicals, a high spontaneous yield of a compound with an energy-rich thioester bond might seem surprising, but the result is expected from thermodynamics because even to the level of the energy-rich thioester, the reaction3.1
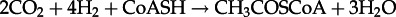
is highly exergonic with *ΔG*°′ = –59 kJ mol^−1^ [72], whereby the pathway becomes endergonic at low H_2_ partial pressures, with *ΔG*°′ = +29 kJ mol^−1^ at approximately 10 Pa H_2_ [73]. Equation (3.1) entails the thiol group of coenzyme A (CoASH) rather than CH_3_SH, and it is the line reaction of the acetyl-CoA or Wood–Ljungdahl [74,75] pathway of microbial CO_2_ fixation. Such favourable energetics led Shock *et al.* [29, p. 73] to conclude that organisms using the acetyl-CoA pathway (acetogens, methanogens and many sulfate reducers) get ‘a free lunch that they are paid to eat’. Provided that transition metals in a hydrothermal vent could catalyse similar reactions as in the laboratory [51], then one would have, in principle, a route for sustained geochemical synthesis of acetyl thioesters (among many other products). That would constitute a critical/crucial link between geochemistry and biochemistry, because acetyl thioesters (acetyl-CoA) are the most central compounds in all of metabolism, as biochemical pathway maps will attest; and apart from the Calvin cycle, which is obviously a late evolutionary invention [76], acetyl-CoA is the direct product of all known CO_2_ fixation pathways [73]. The centrality of acetyl thioesters in modern metabolism is, we contend, a relic of their role in the chemical events that gave rise to metabolism.

Acetyl thioesters are furthermore the most probable entry point of phosphate into metabolism [42,77,78]. In a plethora of microbes, acetyl-CoA is enzymatically cleaved via phosphorolysis to yield acetyl phosphate [79,80], which, with its high free energy of hydrolysis (*ΔG*°′) of –43 kJ mol^−1^, can phosphorylate any number of substrates, including ADP to generate ATP, with a free energy of hydrolysis (*ΔG*°′) of –31 kJ mol^−1^. Weber [81] reported the non-enzymatic synthesis of acyl phosphates from a thioester in the presence of phosphate during the synthesis of pyrophosphate from thioesters, although the acyl phosphate itself was not isolated. A high free energy of hydrolysis of the acyl phosphate bond and simple phosphorylytic synthesis from thoiesters make acyl phosphates good candidates for the first universal currency of high-energy bonds [42], the precursors of ATP.

## 4. Antiquity of the acetyl-CoA pathway

**Figure 2.**
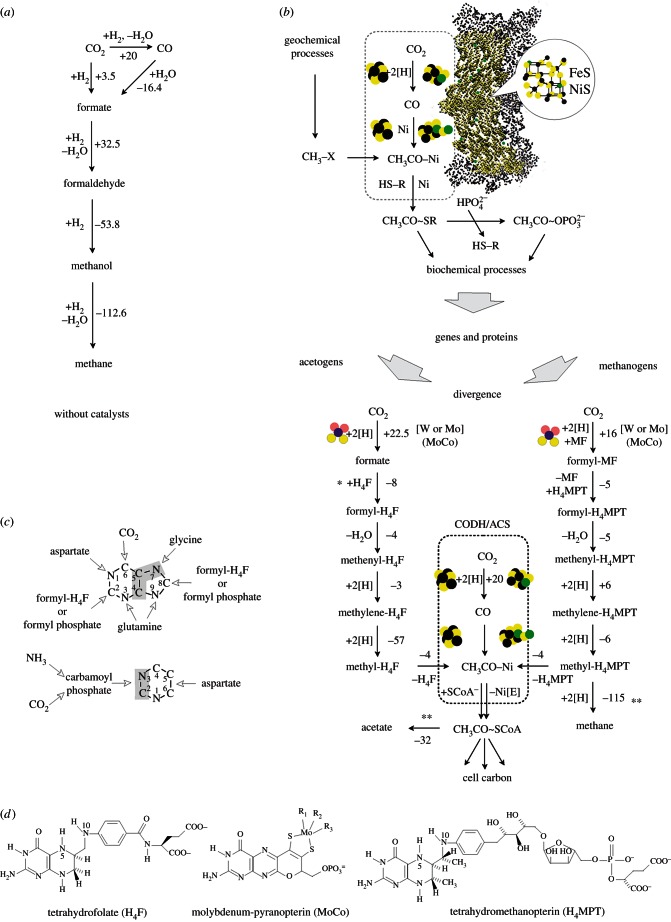
(*Opposite*.) Illustration of some concepts relevant to this paper. (*a*) Abiotic methane production. Summary representation of the H_2_-dependent conversions of CO_2_ to methane without catalysts, adapted from [82]. Numbers next to arrows indicate the approximate change in free energy, *ΔG*°′, for the step indicated at physiological conditions (25°C and pH 7) in kJ mol^−1^, conditions which do not generally exist in geochemical environments. Note, however, that Lost City does produce methane of abiotic origin [20]. The thermodynamic values are taken from Maden [83] and Rother & Metcalf [84]. Regarding the equilibria between the different C_1_ species in the absence of catalysts, see Seewald *et al.* [82]. (*b*) Scheme suggesting homology between the acetyl-CoA pathway in modern acetogens and methanogens to geochemical processes in the hydrothermal vent of a serpentinizing system in the crust of the ancient Earth. See text. Numbers next to arrows indicate the approximate change in free energy, *ΔG*°′, for the step indicated at physiological conditions (25°C and pH 7) in kJ mol^−1^ as reported in [73]. An asterisk indicates ATP investment, a double asterisk indicates ATP return. Note that net ATP return in acetogens and methanogens requires chemisosmotic coupling. FeS and FeNiS clusters are symbolized. Fuchs [73] gives the free energy change for the CODH/ACS reaction as *ΔG*°′ = 0 kJ mol^−1^. The thermodynamic value for the methane-producing step is from [85]. H_4_F, tetrahydrofolate; MF, methanofuran; H_4_MPT, tetrahydromethanopterin; Ni(E), an Fe–Ni–S cluster in CODH/ACS; HSCoA, coenzyme A. For the acetyl-CoA pathway, see also Bender *et al*. [60], Ragsdale [56] and Fuchs [73]. The formate to formyl-H_4_F conversion in acetogens entails ATP hydrolysis (not shown), which lowers *ΔG*°′ for the reaction to –10 kJ mol^−1^. Though all reactions shown are reversible, arrows are shown in one direction only for convenience. (*c*) The source of carbon and nitrogen atoms in the purine and pyrimidine backbone. Data from Stryer [86] and from Ownby *et al*. [87]. (*d*) Pterin cofactors involved in the WL-pathway: MoCo [59] folate and methanopterin [85].

Provided there was a sustained geochemical source of chemically accessible methyl groups (e.g. methyl sufide), the first reactions relevant to the origin of carbon and energy metabolism might have been very similar to those catalysed by the bifunctional enzyme carbon monoxide dehydrogenase/acetyl-CoA synthase (CODH/ACS; figure 2*b*). The catalysis in CODH/ACS is performed by transition metals (Fe and Ni) coordinated as sulfide clusters [50,56,73]. In the enzymatic mechanism [56], electrons from a low-potential Fd are used by the enzyme to reduce one molecule of CO_2_ to CO at an FeNiS cluster called the C cluster; that CO migrates to a second FeNiS cluster in the enzyme (the A cluster), where it binds one of two Ni atoms at the Ni_p_ site to form a Ni-bound carbonyl group, where the two-electron donating property of Ni is important in the catalytic mechanism [56]. A methyl group, donated by the corrinoid iron–sulfur protein CoFeSP, binds the same Ni atom [56] in an unusual metal-to-metal methyl transfer reaction [88], and the carbonyl group inserts the Ni–CH_3_ bond to generate a Ni-bound acetyl group, which is then removed from the enzyme via thiolysis by CoASH to generate the thioester. Given the methyl group, this route of thioester synthesis involves only metals as catalysts and electron donors, no phosphate is involved, and given CO, the reaction works *in vitro*, without enzymes, using either Fe^2+^ or Ni^2+^ as catalysts [70].

CODH/ACS thus has hallmarks of a very ancient reaction, one that could well have proceeded readily before the advent of genes and enzymes. Across the divide that separates acetogens and methanogens, this primordial chemistry at CODH/ACS is homologous and conserved, as is the CoFeS protein [83], but the synthesis of the methyl group is fundamentally different [73], involving distinct, unrelated enzymes and different cofactors—tetrahydrofolate (H_4_F) and tetrahydromethanopterin (H_4_MPT)—both of which are, however, pterins (figure 2*d*), and chemically more similar than the different names suggest [85].

In acetogens, methyl synthesis entails reduction of CO_2_ to formate via an NAD(P)H-dependent formate dehydrogenase, an ATP-consuming step at the 10-formyl-H_4_F synthetase reaction, water elimination via 5,10-methenyl-H_4_F cyclohydrolase, reduction with NADH by 5,10-methylene-H_4_F dehydrogenase, and Fd-dependent reduction via 5,10-methylene-H_4_F reductase to yield 5-methyl-H_4_F which donates the methyl group to corrinoid iron–sulfur protein (CoFeSP) [73]. Of energetic consequence, the formation of the formyl pterin formyl-H_4_F is endergonic, requiring ATP hydrolysis, while the 5,10-methylene-H_4_F reductase step is highly exergonic (–57 kJ mol^−1^) [73] and is suspected to be a site of energy conservation in some acetogen species [89].

In methanogens, the synthesis of the methyl group involves different enzymes and to some extent different cofactors [83]. The first step is the formation of the carbamate formyl-methanofuran (formyl-MF) from CO_2_ and the primary amine of methanofuran. Notably, this reaction is spontaneous, requiring only the cofactor and the substrate, with no help from enzymes [90]. Fd-dependent formyl-MF dehydrogenase and a formyl tranferase yield the formyl pterin formyl-H_4_MPT. A cyclohydrolase, F_420_-dependent 5,10-methylene-H_4_MPT dehydrogenase and F_420_H_2_-dependent 5,10-methylene-H_4_MPT reductase yield methyl-H_4_MPT which donates the methyl group to CoFeSP in carbon metabolism, involving CODH/ACS. In energy metabolism, methyl-H_4_MPT donates its methyl group to coenzyme-M (CoM).

Thus, the reactions at CODH/ACS are catalysed by homologous enzymes, and the methyl-carrying corrinoid protein CoFeSP is homologous in acetogens and methanogens. But in the methyl synthesis branch of the two groups, the only homologous enzymes are at the CO_2_-reduction step. The MoCo-binding subunits of Mo-dependent formyl-methanofuran dehydrogenase, FmdB, and of the W-dependent enzyme, FwdB, in methanogens are related to formate dehydrogenase in acetogens [91], which also exist as Mo- or W-dependent enzymes [92]. The other enzymes are not related, use different cofactors, and in some cases catalyse different reactions. Thus, while it is generally agreed that the acetyl-CoA pathway is the most ancient among modern CO_2_-fixing pathways, the methyl synthesis branches of the Wood–Ljungdahl pathway in acetogens and methanogens are unrelated, even though the methyl groups serve a homologous function at a homologous enzyme, CODH/ACS, en route to thioester synthesis. This suggests three things: (i) the gene for CODH/ACS was present and functioning in their non-free-living common ancestor, (ii) the genes and proteins for the methyl synthesis pathways of acetogens and methanogens arose independently and subsequently to divergence between the archaebacterial and eubacterial stem lineages within the hydrothermal mound, and (iii) that a continuous and abundant supply of accessible methyl groups stemming from geochemical processes—either serpentinization or geochemical redox processes occurring directly at the vent–ocean interface [43]—served as the source of biochemical methyl moieties prior to the advent of the genes and proteins that underpin the methyl synthesis branch of the Wood–Ljungdahl pathway in acetogens and methanogens. This is another way of saying that when the enzymes of methyl synthesis arose, they did not invent methyl synthesis, they just helped chemical reactions that tended to occur anyway, to occur more rapidly.

In this view, the thermodynamically favoured reaction of H_2_ and CO_2_ to methyl groups and methane, similar to that observed in the laboratory [93] or at Lost City [19], was taking place spontaneously (figure 2*a*), and the genes and proteins (figure 2*b*) just helped speed it along and add specificity to a highly selectable function (accelerated self-synthesis, given the necessity of methyl groups for self-synthesis). However, those genes arose in independent stem lineages, from which it follows that methyl groups, whether formed by serpentinization or in the walls of the mound, were abundant at the mound over geological periods of time.

If the reader will grant us, for the purpose of argument, a sustained source of acetyl thioesters at a hydrothermal vent setting, the spontaneous and continued accumulation of organic compounds, including peptides, would be thermodynamically possible. A reaction of this type is what Morowitz [45,94] has, rightly, always demanded. Thioesters could lead to some simple carbon chemistry as outlined in fig. 6 of Fuchs [73], some transition metal catalysed reductive aminations of 2-oxo acids as outlined by Huber & Wächtershäuser [95], and some peptide synthesis aided by transition metals and simple reactive compounds such as carbonyl sulfide [96,97]. Keeping in mind that the pyruvate-synthesizing enzyme of acetogens and methanogens, pyruvate synthase, is replete with FeS centres and uses acetyl-CoA, reduced Fd (Fd_red_) and CO_2_ as substrates [60], and also that reducing conditions of hydrothermal vents make energetics work in favour of amino acid and peptide synthesis [14], an alkaline hydrothermal vent would, in principle, be working in the right direction thermodynamically, because cells consist mainly (approx. 60% dry weight) of protein and to a lesser extent (approx. 25% dry weight) of nucleic acids.

## 5. RNA and the code arose, but that is not our focus

Our focus here is not the primordial synthesis of bases, nor how bases or nucleosides might react with one another, nor what an RNA world might have looked similar in detail, nor the origin of the genetic code. We are interested in the chemical environment and the energetic processes that might have made those important evolutionary steps possible, as exergonic reactions or, more likely, as side products of a central exergonic reaction involving H_2_–CO_2_ disequilibrium (similar to the case in modern microbial metabolism). Three points can be noted.

First, DNA is synthesized from RNA in metabolism, which has long served as an argument that RNA came before DNA in evolution. By the same reasoning, we infer that amino acids came before RNA in evolution, because in metabolism RNA bases are synthesized from amino acids: glycine, aspartate, glutamine, CO_2_ and some C_1_ units in the case of purines; aspartate, ammonia and CO_2_ via carbamoyl phosphate in the case of pyrimidines (figure 2*c*). Amino acids are thermodynamically favoured over nucleotides and thus more likely to accumulate in larger amounts in a hydrothermal setting anyway [35,36]; similarly in metabolism, some might spill over into RNA-like bases. If an RNA world-like system arose in the same environment where the acetyl-CoA pathway arose—a logical consequence of the idea that the acetyl-CoA pathway is the most ancient pathway of carbon fixation [73]—then the RNA world arose in a chemical environment that was very rich in CO_2_, reactive C_1_ moieties and reactive methyl groups. Two of the five carbon atoms in the purine backbone come from reactive C_1_ moieties: 10-formyl-H_4_F or formyl phosphate [87], and one of the five comes from CO_2_, while one of the four carbon atoms of the pyrimidine backbone comes from CO_2_ via carbamoylphosphate synthase reaction (figure 2*c*).

This might be a biosynthetic fossil, an imprint of the chemical environment in which the genes arose that catalysed the first, genetically specified, enzymatic base biosynthesis. That is, the substrates of the enzymes that catalyse base synthesis were probably in existence before the origin of those genes, and the four carbon atoms that stem from CO_2_ or formyl moieties in all modern purine and pyrimidine bases (figure 2*c*) might be relics of life's autotrophic origin. There are limits as to how far one can go with such reasoning, but it is something to consider.

Second, the RNA world as it is currently construed in the laboratory, operates with pure mixtures of usually four bases [98]. But, in the beginning there is just no way that base synthesis could have been highly specific. Any RNA world that might have really existed must have consisted of an ill-defined mixture of bases synthesized spontaneously without the help of either genes or pure chemicals. The chemical environment central to our considerations here is replete with reactive C_1_ moieties and methyl groups (on their way to becoming methane, acetyl groups and acetate); modern ribosome–tRNA interactions at the heart of the genetic code require myriad base modifications in tRNA and rRNA [99], the vast majority of which are methylations [100]. This possible significance of tRNA modifications [101] becomes all the more evident when those common to all prokaryotes that allow the code to function at the wobble base [102] are considered. Or, we can just look at the nature of the chemical modifications that are present in the 28 modified bases common to archaebacteria and eubacteria [100]. They include 19 methyl-, one acetyl-, five thio-, two methylthio-, one seleno-, two methylaminomethyl- and four carbamoyl-moieties [103]. Those modifications might be a chemical imprint—a molecular fossil—of the environment in which tRNA–rRNA interactions (genes and proteins) arose that is preserved in the chemical structure of modified tRNA and rRNA bases. Assuming that any RNA world-like reaction nexus ever really existed in early evolution (a reasonable premise), it would by necessity have consisted of spontaneously and unspecifically synthesized mixtures of bases. The substantial number of enzymes that life forms have carried around for almost 4 billion years to perform these cumbersome and specific modifications, suggests that the modifications are essential, otherwise they would have been discarded. That those enzymes recreate the ancestral state of the RNA world is something to consider.

Third and finally, there is a rather severe and very general problem for all theories on the origin of life. Namely, before there was any kind of genetic feedback via replicating molecules, whether self-replicating or replicated by auxiliary catalysts, we currently have no option but to accept the premise that some replicating chemical entity, catalytic and able to specify synthesis of self, arose spontaneously, which is theoretically possible in the context of autocatalytic networks [104–106]. Morowitz has observed that the chemistry of life is deterministic in many ways, but that does not mean that its emergence is inevitable, because a number of contingent factors, such as the nature of catalysts present, are involved

The origin of life is a deterministic event, the result of the operation of the laws of nature of a physical chemical system of a certain type. This system evolves in time, is governed by physical principles and eventually gives rise to living forms. The details need not be totally deterministic in every respect, but the overall behaviour follows in a particular way. [94, p. 3]

In a world of gene and proteins, evolution via variation and selection can readily supplant chemical determinism. But until we get to things that evolve via variation and selection, all we have are spontaneous reactions under thermodynamic and kinetic control. In this way, thermodynamics is very much chemistry's deterministic version of natural selection [107]. Getting from rocks and water to genuinely evolving systems remains a big problem. But energetics and thermodynamics can help a lot, because they place rather narrow contraints on which paths chemistry can traverse en route in evolving systems that ultimately spawn free-living cells.

That bottom-up perspective has a top-down corollary [108]. If we search among modern microbes and their metabolism for ancient physiological phenotypes, the acetogens and methanogens stand out [44,109]. These obtain their energy from making organic compounds out of CO_2_. In fact, they are often diazotrophic and as such they just live from gases: CO_2_, CO, H_2_, N_2_ and H_2_S. At the level of overall chemical similarity, core carbon and energy metabolism in methanogens and acetogens has quite a lot in common with geochemical processes at alkaline hydrothermal vents [43], and that is probably not coincidence.

Again, we have little to contribute to the nature of an RNA-like world [98], other than the insights that it probably arose in the same environment where the acetyl-CoA pathway did, a setting full of amino acids, reactive C_1_ moieties and CO_2_, and that molecular fossils of that environment might be preserved in RNA base modifications today. Nor do we have anything to contribute to the origin of the genetic code and translation, other than agreeing with our colleagues that it is a very significant and difficult problem [110–112], that it did occur, that the code increased in complexity during evolution [113,114], that the simplicity of the code in methanogens is intriguing [115] and that the origin of the code could have occurred at alkaline hydrothermal vents [116], supported by a continuous flux of harnessable carbon and harnessable energy.

### (a) Chemiosmotic coupling

It should be explicitly stated that, for the purposes of this paper, we are assuming that the origin of genes and proteins could have been fuelled with acyl phosphates (acetyl phosphate) as the central currency of ‘high-energy bonds’, that these were generated by substrate-level phosphorylation via reactions as outlined earlier (figure 2*b*), and that this requires a steady input of spontaneously synthesized methyl groups of geochemical origin [109], either from serpentinization or from redox processes at the vent–ocean interface [43]. The standard free energy of hydrolysis (*ΔG*°′) of acetyl phosphate is –43 kJ mol^−1^, meaning it can phosphorylate ADP to ATP (*ΔG*°′ –31 kJ mol^−1^), and by the same token it can drive phosphorylations in intermediary metabolism as well as ATP does. However, the actual *ΔG*°′ provided by a reaction depends on the level of disequilibrium between rates of formation and breakdown—specifically the steady-state ratio of acetyl thioesters to acetyl phosphate to acetate, which is ultimately driven far from equilibrium by the chemical disequilibrium generated by serpentinization.

Once increasing chemical complexity transforms into conventional evolution of genetically encoded proteins, the invention of new functions potentially becomes a very fast process. The jump we just took, to genes and proteins, is a big one, but all models for the origin of life entail such a step, and ours does too. Recalling that its mechanisms are, as stated earlier, not our focus, we proceed. At some early point, the advent of ATP as the universal energy currency was an important step in bioenergetic evolution, displacing (we posit) acetyl phosphate. However, while ATP is universal across lineages, it is not the sole energy currency within the metabolism of individual cells by any means [80]. This is an interesting and possibly significant point, suggesting that much went on in early biochemistry before ATP became a common currency. The simplest explanation for ATP's rise to prominence is that it was a consequence of the substrate specificity of the rotor–stator-type ATPase, a protein that is as universal among cells as the code, and that is unquestionably an invention of the world of genes and proteins [42]. Given genes and proteins, the origin of molecular machines such as the ATPase is an admittedly impressive, but not conceptually challenging, evolutionary step; it is a far less problematic increment than the hurdles that had to be surmounted at the origin of the ribosome and the code [110,117].

In an alkaline hydrothermal environment, the ATPase has immediate beneficial function. The naturally preexisting proton gradient [9] at the interface of approximately pH 10 alkaline effluent (alkaline from serpentinization) with approximately pH 6 ocean water (slightly acidic by virtue of high CO_2_ content) provides a geochemically generated chemiosmotic potential that ‘just’ needs to be tapped, that is, converted into chemically harnessable energy in the form of high-energy bonds, exactly what rotor–stator-type ATPases do. That would have put a very high-energy charge on the contents of the inorganic compartments within which we presume that this was all taking place, accelerating biochemical innovations, energetically financing gene inventions, and the selective pressure on evolving proteins to adapt to a new energy currency is evident. ATP-binding domains are so prevalent in genomes [118], not because ATP is a constituent of RNA, but because it became the most popular energy currency. The ATPase transduced a geochemically generated ion gradient into usable chemical energy, and since the energy was free, the means to harness it as ATP ‘just’ required a suitable protein for the job, a complicated protein [119], but a protein.

One might object that the multi-subunit rotor–stator-type ATPase is too complicated as a starting point, and that it is hence preferable to assume that some simpler form of energy transducing protein, for example, a pyrophosphatase, preceeded it [120]. But the observation from modern microbes to be accounted for, in the evolutionary sense, is that all prokaryotes harbour the rotor–stator-type ATPase. Similar to a handful of only 30 or so other proteins, it is as universal as the ribosome; hence it was probably present in the common ancestor of all cells, which in this model was not free-living but rather was confined to the system of inorganically formed compartments within which it arose. Consistent with that, prokaryotic ATPases show a clear dichotomy into two types: the eubacterial (or F-type) ATPase and the archaebacterial (or A-type) ATPase [121]. That dichotomy is also mirrored by a number of other cellular, molecular and gene homology attributes, indicating that the deepest, most ancient split in the prokaryotic world is that separating eubacteria from archaebacteria [111,116,122].

That suggests that the last universal common ancestor (LUCA), which we posit to be a geochemically confined ancestor (figure 1), possessed among other things genes, proteins, the code, along with biosynthetic pathways for amino acids, bases and cofactors to support functional ribosomes and an ATPase that could tap the naturally chemiosmotic gradient at the vent–ocean interface. But it was far short of being a free-living cell. Many attributes of archaebacteria and eubacteria are so fundamentally different that, for lack of similar chemical intermediates in the pathway or for lack of subunit composition or sequence similarity, independent origin of the genes underlying those differences is the simplest explanation. Such differences include: (i) their membrane lipids (isoprene ethers versus fatty acid esters) [123], (ii) their cell walls (peptidoglycan versus S-layer) [124], (iii) their DNA maintenance machineries [116,125], (iv) the 31 ribosomal proteins that are present in archaebacteria but missing in eubacteria [126,127] (v) small nucleolar RNAs (homologues found in archaebacteria but not eubacteria) [128], (vi) archaebacterial versus eubacterial-type flagellae [129], (vii) their pathways for haem biosynthesis [130,131], (viii) eubacterial- versus archaebacterial-specific steps in the shikimate pathway [132,133], (ix) a eubacterial-type methylerythrol phosphate isoprene pathway versus an archaebacterial-type mevanolate isoprene pathway [134], (x) a eubacterial-type fructose-1,6-bisphosphate aldolase and bisphosphatase system versus the archaebacterial bifunctional aldolase-bisphosphatase [135], (xi) the typical eubacterial Embden–Meyerhoff  (EM) and Entner–Doudoroff  (ED) pathways of central carbohydrate metabolism versus the modified EM and ED pathways of archaebacteria [136], (xii) differences in cysteine biosynthesis [137], (xiii) different unrelated enzymes initiating riboflavin (and F_420_) biosynthesis [138], and (xiv) in very good agreement with figure 2*b*, different, unrelated, independently evolved enzymes in core pterin biosynthesis [139], to name a few examples. The pterin biosynthesis example is relevant because the cofactors H_4_F, H_4_MPT and MoCo, which are central to the eubacterial and archaebacterial manifestations of the Wood–Ljungdahl pathway are pterins (figure 2*d*), suggesting that methyl synthesis occurred geochemically (non-enzymatically) for a prolonged period of biochemical evolution.

**Figure 3.**
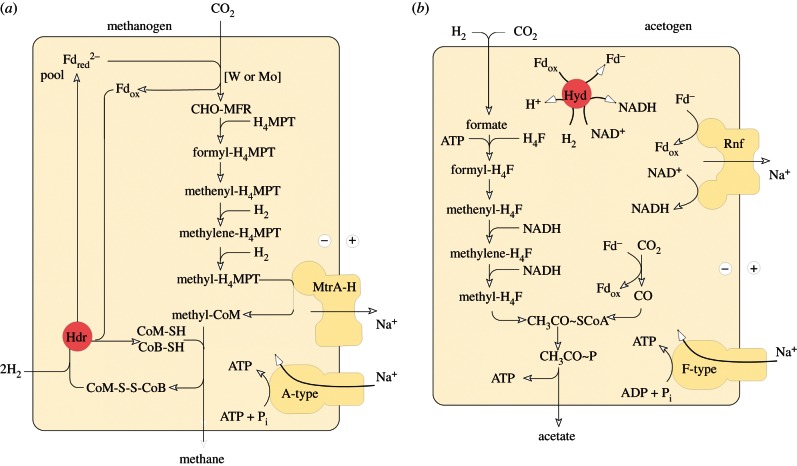
Energy metabolism of (*a*) methanogens and (*b*) acetogens without cytochromes. Redrawn for *Methanothermobacter marburgensis* from Thauer *et al*. [40], Kaster *et al*. [145] and Thauer & Buckel [143], redrawn for *Acetobacterium woodii* from Pohlein *et al*. [146] and Thauer & Buckel [143]. When we refer to acetogens and methanogens that lack cytochromes, we are referring to the physiology in those organisms, as the examples. The use of the symbol Fd^2–^ indicates that the ferredoxin in question has two FeS centres, both of which become reduced. MtrA-H, methyl transferase complex [40]. Rnf: Fd:NADH oxidoreductase, originally named for *Rhodobacter* nitrogen fixation [147]. Other abbreviations as in figure 2. Enzymes known to perform electron bifurcation are indicated in red: heterodisulfide reductase (Hdr) [145] and hydrogenase (Hyd) [148].

It has been argued that these deep differences between archaebacteria and eubacteria reflect comparatively recent environmental adaptations; a specific claim is that archaebacteria adapted very recently [140]. However, many eubacteria thrive in hyperthermophilic environments without going to the trouble of replacing all their membranes and walls, DNA replication, etc., while plenty of archaebacteria thrive in mesophilic environments such as the open oceans and soils. It is therefore not the case that archaebacteria are somehow better adapted to hyperthermophilic conditions whereas bacteria are better adapted to mesophilic conditions, even though the perception of archaebacteria as extremophiles persists. Alternatively, the differences of archaebacteria and eubacteria have been interpreted as adaptations to energy stress [141], but both acetogens and methanogens live at the lower end of the free energy spectrum whereby acetogens live from less energy than methanogens [142,143] but without having become archaebacteria, so the energy stress argument is clearly not robust. Given the almost equally wide environmental representation of both groups, both in energy-rich and in energy-poor environments [144], the deep divergence between them in all likelihood simply reflects the very early evolutionary divergence, in our view physical divergence, of replicating compartment contents within the mound that led to two stem lineages which became the common ancestors of eubacteria and archaebacteria, respectively (figure 1). Physiologically, those stem lineages gave rise to acetogens and methanogens. The similarities and differences in carbon metabolism (the Wood–Ljungdahl pathway) in acetogens and methanogens outlined earlier (figure 2) are paralleled by similarities and differences in their mechanisms of energy conservation (figure 3), which entail chemiosmotic coupling.

At first sight, the idea that chemiosmosis is a very ancient means of energy transduction might seem counterintuitive. More familiar to many is the old (and popular) doctrine that the most ancient pathway of energy metabolism is a fermentation such as glycolysis [77], an idea that goes back at least to Haldane [2] and hence arose long before anyone had a clue that biological energy can be harnessed in a manner that does not involve substrate-level phosphorylations and ‘high-energy’ bonds [149,150]. In modern life, all biological energy in the form of ATP comes ultimately from chemiosmotic coupling [151], the process of charge separation from the inside of the cell to the outside, and the harnessing of that electrochemical gradient via a coupling factor, an ATPase of the rotor–stator-type. It was not until the 1970s that it became generally apparent that Mitchell [152] was right, his Nobel prize coming in 1978, and it is hard to say when it became clear to microbiologists that all fermentative organisms are derived from chemiosmotic ancestors. We also note that Mitchell's consideration of the problem of the origin of life introduced key concepts of his later chemiosmotic hypothesis, including a definition of life as process, and the idea of vectorial catalysis across a membrane boundary that is inseparable either from the environment or from the organism itself [153].

The maxim that glycolysis is ancient might be an artefact of experience, since it was the first pathway both to be discovered and that we learned in college; in that sense, it really is the oldest. When one suggests that chemiosmotic coupling in methanogens or acetogens might be ancient, many listeners and readers shy away, mainly because the pathways are unfamiliar and often entail dreaded cofactor names. The familiar cytochrome complexes, quinones and quinone-based charge translocation are lacking in these apparently ancient life forms, which possess no complexes I, II or III, no terminal oxidases, and only a single coupling (ion pumping) site each. Their energy metabolic pathways differ so fundamentally from the familiar respiratory chain-type chemiosmotic pathways of *E. coli* or mitochondria that only recently have biochemists uncovered how their basic energetics works. Yet, despite these fundamental differences, and their unfamiliarity to most of us, the bioenergetics of methanogens and acetogens nonetheless feature bona fide chemiosmotic circuits, with coupling proteins, ion electrochemical gradients over membranes and the ATP synthase. These pathways are illustrated in figure 3, which recapitulates the schemes presented by Buckel & Thauer [143], Thauer *et al.* [40] and Poehlein *et al.* [146], for methanogens and acetogens that lack cytochromes. Acetogens and methanogens are unique among chemiosmotic organisms studied so far in that both types exist as forms that lack cytochromes and quinones.

In brief, methanogens that lack cytochromes, as *Methanobacterium marburgensis* (figure 3*a*) use their version of the Wood–Ljungdahl pathway to synthesize methyl-H_4_MPT, but instead of donating it to CoFeS and CODH/ACS as in carbon metabolism (figure 2*b*), they donate it to the thiol moiety of CoM in an exergonic reaction (*ΔG*°*‘* = *ca* –30 kJ mol^−1^) catalysed by a membrane-bound methyltransferase (MtrA-H in figure 3*a*), which conserves energy via the pumping of Na^+^ ions across the cytoplasmic membrane [40]. CoM-SH is regenerated from methyl-CoM by methyl-CoM reductase, which releases methane by condensing the CoM moiety with the thiol group of coenzyme B (CoB-SH) producing the heterodisulfide CoM-S-S-CoB. The CoM-SH and CoB-SH thiols are regenerated by reduction with electrons from H_2_ by a heterodisulfide reductase (Hdr in figure 3*a*), in a highly exergonic reaction (*ΔG*°*‘* = –55 kJ mol^−1^) [145]. Hdr also generates Fd_red_, which serves as the low-potential reductant for the formyl-MF dehydrogenase step at the beginning of the WL-pathway, and the significance of this Hdr reaction will be discussed shortly. Per mol of methane, 2 mol Na^+^ ions are pumped, whereas the ATPase requires about 4 Na^+^ per ATP [145]. The overall reaction is that given in reaction (2.4), whereby, per mol methane, the cell is able to condense about 0.5 mol of ATP from ADP and P_i_ [145]. Aside from the anhydride bond in the ATP that is the product of the pathway, there are no thioesters, no acyl phosphates and no ATP consumption involved in methane production. The main energy currency is low-potential-reduced ferredoxin, Fd_red_ [143].

Acetogens that lack cytochromes use their version of the WL-pathway to make methyl-H_4_F, as sketched in figure 3*b*, which is modified from Pohlein *et al.* [146] and Buckel & Thauer [143] for *Acetobacterium woodii*. ATP investment (a concept familiar from glycoysis) is required at the endergonic formyl-H_4_F synthetase step, formyl phosphate (figure 2*c*) being the ‘activated’ ATP-generated reaction intermediate, both in *E. coli* [154,155] and in the acetogenic enzyme [156]. The methyl group of methyl-H_4_F is transferred via CoFeSP to CODH/ACS where the acetyl-CoA is released. The energy in the thioester bond is conserved as acetyl phosphate in the phosphotransacetylase reaction. Acetate kinase converts acetyl phosphate and ADP into acetate and ATP, thereby recovering the ATP investment at the formyl-H_4_F synthetase step. Methyl synthesis consumes electrons from H_2_ according to reaction (2.3). The *A. woodii* formate dehydrogenase probably uses H_2_ directly as substrate [146], whereas the remaining two reduction steps are NADH-dependent [143,146]. The NADH stems from a soluble hydrogenase (Hyd) that, importantly, also generates Fd_red_ in the process [148]. Fd_red_ is the substrate for a membrane-bound protein called Rnf [147] that generates NADH and pumps, in the process, about one Na^+^ ion per electron transferred, thus generating the ion gradient that is harnessed by the ATPase, which requires about 4 Na^+^ ions per ATP. Per mol of acetate, about 0.25 mol of ATP is generated [143]. The ATP investment at formyl-H_4_F synthetase step and return via acetate kinase is strictly 1 : 1, so there is no net gain of ATP via that route. The currency of energy conservation is again Fd_red_, which powers pumping at Rnf to drive the ATPase.

## 6. Electron bifurcation

Both acetogens and methanogens reduce CO_2_ to a methyl group using electrons from H_2_. This reaction actually should not proceed at equimolar concentrations, because the midpoint redox potential, *E*°′, of the H_2_/H^+^ couple is –414 mV, while the *E*°′ for the CO_2_/formate couple is –430 mV and for the formate/formaldehyde couple it is –580 mV. In simple terms, H_2_ is not a strong enough electron donor to reduce CO_2_ to the level of a methyl group; the electrons would have to go steeply uphill. This prompted Wächtershäuser [157, pp. 1790–1791] to conclude that H_2_ can be ‘excluded as the first source of electrons since its reducing potential is not sufficient for reducing CO_2_’. So how do chemolithoautotrophs reduce CO_2_ with electrons from H_2_? The simple answer would be that they generate low-potential Fd_red_ with midpoint potentials of the order of –500 mV, but that is also still steeply uphill from H_2_, bringing us to the crux of the issue: how do they generate Fd_red_ from H_2_? The answer, which only recently became apparent and is an exciting development in energetics, is that they perform a reaction called flavin-based electron bifurcation [143,158]. This electron bifurcation occurs at the reaction catalysed by Hdr in the methanogens, and at the reaction catalysed by Hyd in the acetogens (figure 3).

**Figure 4.**
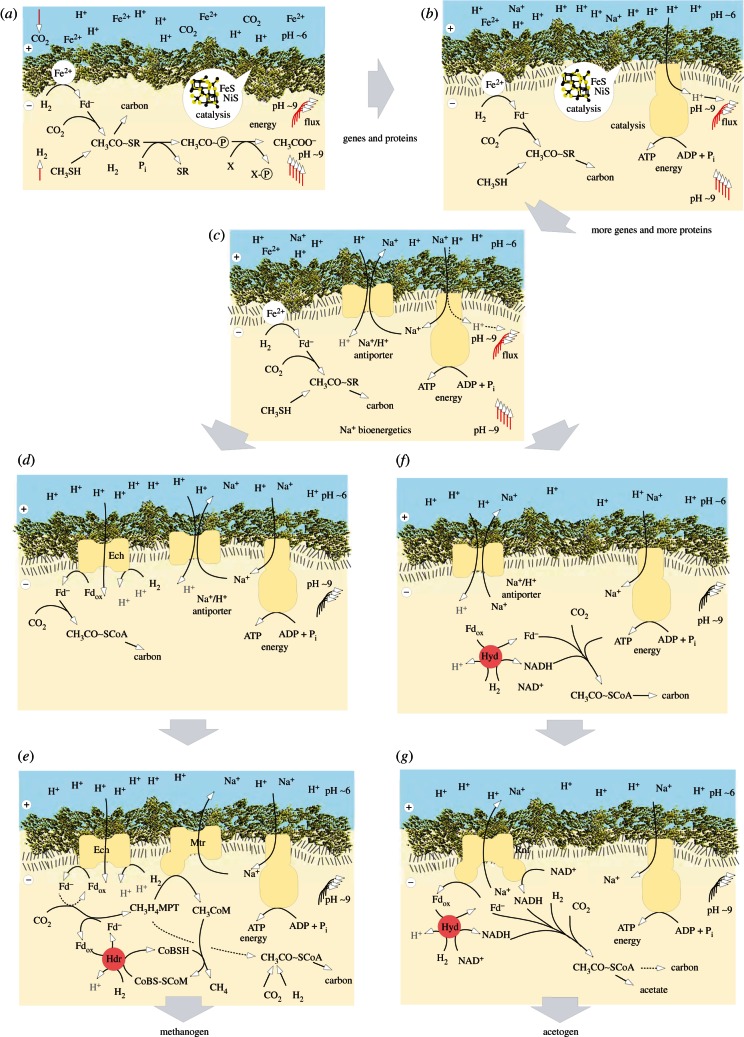
A hypothetical path for the events linking figures 2 and 3, redrawn from [43]. Ech: Energy converting hydrogenase [159]. Other abbreviations as in figures 2 and 3. See text.

This newly discovered mechanism couples an endergonic reaction to an exergonic one, analogously to the familiar case of coupling an unfavourable reaction to ATP hydrolysis so that the overall energetics of the reaction are favourable. But in electron bifurcation, no ATP is involved. Instead, it entails the splitting of electron pairs, from H_2_, for example (*E*°′ = –414 mV), at a flavin, such that one electron goes energetically uphill to generate reduced low-potential ferredoxins (Fd_red_) with a midpoint potential close to –500 mV, with the energy for that uphill climb being provided by the second electron of the pair going downhill to a high-potential acceptor, such as the heterodisulfide CoM-S-S-CoB (*E*°′ = –140 mV) of methanogens [145] or NAD^+^ (*E*°′ = –320 mV) at the electron-bifurcating hydrogenase of acetogens [148]. Electron bifurcation permits conservation of biochemical energy in the currency of reduced ferredoxin—a principle of energy conservation that departs from Lipmann's [149] high-energy bonds. Fd_red_ contains no cleavable ‘high-energy’ bonds, but electrons at low potential, so it is clearly a currency of chemical energy [143]. Oxidation of Fd_red_ by a pumping complex such as Rnf [147] or the Ech hydrogenase [159] couples the electron bifurcation mechanism to chemiosmotic energy conservation (figure 4).

The electrons in low-potential Fd_red_ are crucial for CO_2_ reduction in organisms that use the acetyl-CoA pathway. By splitting the electron pair in H_2_ with one going uphill to Fd the other going downhill to a high-potential acceptor, these organisms can reduce CO_2_ under what might seem to be energetically hopeless conditions. The prevalence of electron bifurcation among strict anaerobes suggests that it is a very ancient biochemical mechanism [73,143], one that was apparently a prerequisite for a lifestyle of reducing CO_2_ with electrons from H_2_, which is how organisms that use the acetyl-CoA pathway for their carbon and energy metabolism survive.

## 7. Counting rocks in genomes

In anaerobes, Fd is usually one of the most abundant proteins in the cell, pointing to the central importance of this small, FeS cluster containing protein in their energy metabolism. But Fd is not the only abundant FeS protein in these organisms. The proteins of anaerobes are generally rich in transition metals and transition metal sulfide clusters at the catalytic centres of their proteins, catalytic clusters which are often replaced by NAD^+^ and other organic cofactors in aerotolerant species. The early Earth was devoid of molecular oxygen [30]; hence the first life was anaerobic. There is a consistent and robust line of biochemical evolutionary reasoning that FeS clusters, chemolithoautotrophy and anaerobes tend to reflect antiquity [44,49,160,161]. That line of reasoning is still current [73,162,163] and is reinforced by geochemical data providing evidence for the antiquity of methanogenesis [164].

**Figure 5.**
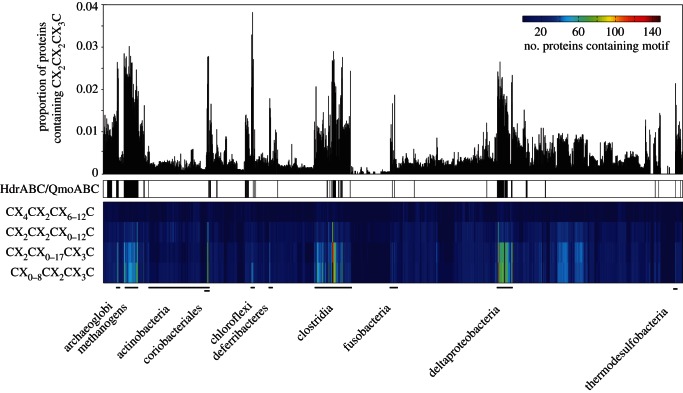
Occurrence of 4Fe–4S cluster motifs among 1606 prokaryotic genomes. The upper part of the figure represents the results of a search for the general form of the 4Fe–4S cluster-forming protein motif CX_2_CX_2_CX_3_C. The proteins of 1606 prokaryotic genomes from the RefSeq database (v03.2012) [166] were under examination for this. The prokaryotes are represented by single bars and are ordered by their taxonomical classification. The height of each bar indicates the proportion of proteins within a single genome containing the motif. The lower part of the figure gives the absolute number of proteins containing one of the four additional motifs, which have different numbers of bridging amino acids. The order of the columns is the same as the corresponding bars in the upper part. The following prokaryotes having a high abundance of CX_2_CX_2_CX_3_C motif containing proteins were marked: archaeoglobi, methanogens (methanobacteria, methanococci, methanomicrobia), coriobacteriales (actinobacteria), dehalococcoidetes (chloroflexi), deferribacteres, clostridia, fusobacteria, deltaproteobacteria and thermodesulfobacteria. All 1606 taxa were also checked for the presence of heterodisulfide reductase subunits (HdrABC) or its relative, the quinone-interacting membrane-bound oxidoreductase subunits of sulfate reducers (QmoABC), as these might hint at the presence of electron bifurcation involving these proteins; black bars indicate taxa where at least one of them was present. Note that several other enzymes known to be involved in electron bifurcation [143] are not indicated.

If FeS clusters are relics from the ancient past, it could be that the most ancient organisms preserved more FeS-containing proteins in their genomes. Major *et al.* [165] found that among the 120 genomes sampled then, the methanogens had by far the highest frequency of FeS proteins. Here, we have updated their survey with 1606 genomes, looking for the frequency of 4Fe-4S clusters of the type CX_2_CX_2_CX_3_C. We observed the distribution shown in figure 5 which clearly shows that methanogens, acetogens and sulfate reducers have the highest frequencies of FeS proteins. This fits well with our general premises, but it does not prove anything. There is a point to be made, though, in that the case has recently been argued that ZnS, rather than transition metal sulfides, might have been the starting point of prokaryotic biochemistry [5]. However, it should be stressed that there is a big difference between the view that life emerged from a ZnS-mediated process and the view that life emerged from FeS and other transition metal sulfide-mediated processes. In the model of Mulkidjanian *et al.* [5], ZnS has the role of providing electrons (photochemically), a role that we ascribe to serpentinization (geochemically), but that is not such a big difference. The big difference is that Zn is a substrate in their model, providing two electrons per atom in the one-off photochemical reaction, not a catalyst (which FeNiS is in our model). Zn is not catalytic in the way that Fe, Ni, Mo, W or other transition metals are anyway, for which reason modern anaerobes lack ZnS centres participating in redox reactions of carbon or energy metabolism. The Zn model lacks redox catalysis and similarity to modern metabolism.

Following the physiological line of reasoning on evolution a bit further, Decker *et al.* [44] suggested that the next physiological group to arise following the acetogens and the methanogens were the sulfate reducers, strict anaerobes, many of which are autotrophs that use the acetyl-CoA pathway [167–171]. This notion would fit very well within the present considerations, including the circumstance that there are archaebacterial and eubacterial sulfate reducers [172]. It is furthermore supported by geochemical isotope evidence that attests to the existence of processes performed by sulfate reducers—sulfate/sulfite reduction and sulfur disproportionation—in the early Archaean era, around 3.4 billion years ago [173–175].

The ancestral metabolism performed by sulfate reducers was likely to have been sulfite reduction or sulfur disproportionation—and perhaps also disulfide disproportionation, as newer findings suggest [176]—since sulfur and sulfite would be produced abundantly from volcanic and hydrothermal SO_2_, whereas sulfate, prior to the advent of oxygen, would probably have had only very limited, localized significance [177]. In line with that, the use of sulfate as a substrate by sulfate reducers involves soluble ATP consumption steps to produce sulfite [172]. On the early Earth, localized sulfate concentrations could be formed abiotically from atmospheric photolysis of SO_2_, or biologically from sulfide-dependent anoxygenic photosynthesis or sulfur disproportionation, but it was not until the increased oxygenation of the atmosphere that oceanic sulfate levels started to increase and sulfate began to play a prominent role in the global sulfur cycle [178]. Dissimilatory sulfite reduction is a more widespread trait than sulfate reduction, and the enzyme responsible for it, the dissimilatory sulfite reductase (DsrAB) is clearly an enzyme of very ancient origin, and it is closely related to the assimilatory enzyme present in all domains of life [179]. Both assimilatory and dissimilatory enzymes result from a gene duplication event that preceded the divergence of the archaebacterial and eubacterial domains [180–182], so a primordial sirohaem-containing sulfite reductase was most probably present in LUCA. Note that the presence of sirohaem does not mean the presence of haem in LUCA, because sirohaem synthesis merely entails the insertion of Fe^2+^ into sirohydrochlorin, an intermediate of anaerobic cobalamine synthesis (which acetogens and methanogens possess) and sirohaem is furthermore a precursor of the archaebacterial (alternative) haem biosynthetic pathway [183].

Something else that speaks rather strongly for the antiquity of methanogens, acetogens and sulfate reducers is their prevalence in the deep biosphere, where, as on the early Earth, there are few ways to make a living. Sulfate reducers, acetogens and methanogens appear to be the most common inhabitants of ecosystems situated deep within the crust [184–188]. They inhabit such environments today, suggesting that their ancestors might have as well. Curiously, though not involved in energy metabolism directly, primitive forms of Dsr-like proteins are apparently abundant in methanogens [189], with a role in sulfite assimilation or detoxification, further stressing the close ties between sulfate reducers and methanogens.

## 8. Electron bifurcation and sulfate reducers

Another link between methanogens and sulfate reducers is the occurrence in the latter of a number of Hdr-related proteins, suggestive of electron bifurcation [172]. For methanogens, the critical bifurcation reaction, described earlier, is catalysed by a complex of a heterodisulfide reductase with a hydrogenase, the MvhADG–HdrABC complex [145]. Homologues of HdrABC are found in other organisms, including at least one cytochrome-containing acetogen, *Moorella thermoacetica* [89], and many proteobacterial and clostridial sulfate reducers [172]. In both cases, it is suspected that HdrABC-mediated electron bifurcation is participating in core carbon and energy metabolism, but this has not been directly shown so far. For *Moorella*, HdrABC homologues are encoded directly next to the gene for methylene-H_4_F reductase, which catalyses a very exergonic reaction and was therefore once suggested as a coupling site for energy conservation [39]. It remains possible that the *Moorella* methylene-H_4_F reducatase does conserve energy, but not via ion pumping as originaly envisaged, rather via electron bifurcation [89] and Fd reduction instead. Recently, an electron-bifurcating ferredoxin- and NAD-dependent [FeFe]-hydrogenase (HydABC) from *Moorella* was shown to catalyse the reversible bifurcation reaction [190]. For the sulfate reducers, several organisms contain HdrABC or HdrA-like proteins, encoded next to genes for possible electron donor proteins such as an MvhADG hydrogenase, a NAD(P)H dehydrogenase or a formate dehydrogenase [172], strongly suggesting that electron bifurcation is also involved in their energy conservation.

Furthermore, several proteins directly implicated in sulfate/sulfite reduction are strikingly related to HdrABC subunits. Sulfate reducers contain menaquinone, but its role in respiration was for long a mystery because the terminal reductases, APS reductase (AprBA) and DsrAB, are soluble cytoplasmic enzymes. A link between the two started to emerge with the identification of two membrane complexes having quinone-interacting subunits, QmoABC and DsrMKJOP, as probable electron donors to AprBA and DsrAB [191–193]. Both these complexes have Hdr-related subunits. The QmoABC complex is essential for sulfate reduction [194], and two of its subunits, QmoA and QmoB, are both flavoproteins closely related to the bifurcating subunit HdrA. In the DsrMKJOP complex, the two DsrMK subunits are closely related to the membrane-associated HdrED enzyme present in cytochrome-containing methanogens. DsrK contains the special catalytic FeS cluster that in HdrB is responsible for reduction of the heterodisulfide, which in the case of the sulfate reducers is proposed to be the small protein DsrC that is involved in sulfite reduction by DsrAB [195].

How energy is conserved by the QmoABC and DsrMKJOP membrane complexes has not been clearly established, but a recent proposal suggested a new mechanism of electron confurcation—bifurcation in the reverse direction [196]—involving menaquinone in the case of Qmo [197]. In this proposal, the endergonic reduction of APS by menaquinol is coupled to its exergonic reduction by a low-potential soluble electron donor (such as Fd). In contrast to electron bifurcation where a single-electron donor (NADH, H_2_ or formate) is used to reduce two electron acceptors, one of high and one of low potential (most commonly Fd), in this quinone-linked confurcation two electron donors, of high (menaquinone) and low potential (Fd?), are used to reduce a single-electron acceptor. Soluble confurcation processes have already been described, such as those involving multimeric hydrogenases (NADH and Fd_red_ as electron donors for H_2_ production) [196] and the NfnAB transhydrogenase (NADH and Fd_red_ as electron donors for NADP^+^ reduction) [89,198]. Having menaquinone as one of the confurcation players would allow for transmembrane electron transfer and charge separation to effectively couple the process to chemiosmotic energy conservation. This could not occur if only a soluble low-potential donor was involved. It is possible that this, so far speculative, idea might also apply to other anaerobes for which the role of menaquinones is so far unclear, and where MK does not have a sufficiently low reduction potential to participate in core energy or respiratory metabolism. The cytochrome-containing acetogens such as *Moorella*, in particular, come to mind in this respect, because they contain menaquinone, but there is yet no obvious role for it in core metabolism [89].

The involvement of electron bifurcation in sulfate reducers points further to the antiquity of sulfate/sulfite reduction as an ancient anaerobic physiology and is consistent with the observation that a number of sulfate reducers use the acetyl-CoA pathway [199–201].

## 9. From harnessing to pumping: the same leap by two prokaryotes

Electron bifurcation in methanogens and acetogens permits generation of membrane potential from H_2_ and CO_2_, which can then be used to drive net ATP synthesis via the ATP synthase, or in the case of *M. marburgensis*, to reduce ferredoxin via the energy converting hydrogenase, Ech. Thus, electron bifurcation helps cells generate what alkaline hydrothermal vents geochemically provide for free: ion gradients. Given the foregoing, the continuous reduction of CO_2_ to organics could have ultimately driven the emergence of RNA, proteins and genes within the vent pores: what we shall call protocells, meaning the organic contents of inorganic pores within the microporous labyrinth of alkaline hydrothermal vents, but not, as yet, fully functional or independently dividing cells. How (and why) did these confined protocells, dependent on natural pH gradients, begin to generate their own gradients, enabling them ultimately to leave the vents as self-sufficient, free-living cells?

One possible solution depends on the counterintuitive properties of membranes in the presence of natural ion gradients [43]. Apart from the remarkable sophistication of modern respiratory chains, a second reason that chemiosmotic coupling is usually considered to have arisen late relates to the permeability of early membranes. Chemiosmotic coupling today requires an ion-tight membrane, so that ions pumped out return mostly through the ATP synthase, driving ATP synthesis. If ions can instead return through the lipid phase of a leaky membrane, ATP synthesis is said to be uncoupled—literally, short-circuited—and most of the energy consumed in pumping is simply dissipated as heat. Thus, pumping protons across a leaky membrane costs a lot more energy than can be conserved as ATP, making it worse than useless. The problem at the origin of life is that early membranes would certainly have been leaky, hence pumping protons across them could offer no advantage, especially in the presence of small organic acids which naturally dissipate proton gradients. Pumping protons at the vent is pointless anyway, as the proton gradient already exists. A second related problem is that archaebacteria and eubacteria do not have homologous membranes; hence we have argued that their last ancestor, confined to vents, did not have a modern membrane. While simple phase separation of amphiphiles, fatty acids, alkanes and other hydrophobics would tend to produce hydrophobic layers within the vent pores, probably even lipid bilayers, these were likely to be very leaky to small ions.

But arguments against early chemiosmotic coupling share a common flaw: they assume that pumping arose before harnessing. In the presence of natural proton gradients, this is not the case. We have argued that the ATPase arose before pumping mechanisms. The passage of protons through the ATP synthase today is about 4–5 orders of magnitude faster than through the lipid phase of the membrane. But with a free, geochemically provided gradient, membrane permeability could be orders of magnitude greater, while still allowing net ATP synthesis. All that is required is that protons should pass through the ATP synthase as well as through the lipid phase (and that they be removed by the alkaline effluent). Any improvements in the ion-tightness of the membrane would therefore be beneficial, as they would funnel a larger proportion of protons through the ATPase. Hence if there were any genetic component to early membranes, we would predict that coupling would steadily improve, which is to say that organic membranes would tend to become less permeable to small ions over time. On the face of it, that seems a reasonable statement, but it leads to an unexpected problem.

The problem relates to the transfer of charged particles—protons—across the membrane. In vents, neither hydrothermal fluids nor ocean waters carry a net charge: the excess of H^+^ in the ocean is balanced by HCO_3_^–^, whereas the excess of OH^–^ in hydrothermal fluids is balanced by Mg^2+^. Thus, the transfer of a single proton across the membrane down a concentration gradient also transfers a positive charge, and this opposes the further transfer of charge—which is to say, more protons. Soon, a Donnan equilibrium is established, in which the electrical charge across the membrane balances the concentration gradient, annulling the proton-motive force. This problem is only unmasked if the membrane can actually hold an electric charge, which is to say, only if the membrane has become impermeable to small ions. Before that, however—if the membrane is leaky or discontinuous—the system is essentially open, and charge does not enter into it. Any H^+^ crossing the membrane will react with hydrothermal OH^–^ to form water, which is swept away in the flow, negating the accrual of charge. In such an open system, the pH gradient is maintained not by pumping, but by the continuous circulation of hydrothermal fluids and ocean waters, continually juxtaposing phases in redox and pH disequilibrium. Thus, at an early stage, the proton-motive force can only exist in the presence of leaky membranes and an open system: far from being a problem, leakiness is actually a necessity.

What happens when the membrane tightens off to small ions and forms cell-like structures? Transfer of protons into an impermeable cell will result in a Donnan equilibrium that opposes further transfer unless counterions are also transferred, or unless the proton can be pumped out again. Pumping out a proton is problematic. Either the cell needs to invent machinery to pump out protons as soon as the membrane becomes impermeable (plainly impossible) or it needs to invent this machinery while the membrane is still permeable, i.e. before it needs it. This is also unlikely, as the cell would need to actively pump protons (costing energy) from an internal concentration of pH 9–10 (less than or equal to 1 nM) across a leaky membrane against a 1000-fold concentration gradient, which is immediately dissipated through the leaky membrane—a common objection to chemiosmotic coupling evolving early. The solution is surprisingly simple: a Na^+^/H^+^ antiporter, an otherwise unassuming protein that would hardly be a prime suspect for evolutionary heroics, except in alkaline hydrothermal vents.

In a world of genes and proteins, a Na^+^/H^+^ antiporter is far easier to invent than an ATPase. It is a single protein, and an ancient one that is also a component of both Ech and Complex I [202,203]. But it confers an inordinate benefit. An antiporter transfers a positive charge in the opposite direction to the proton, meaning that overall the passage of a proton down a concentration gradient is charge neutral. Thus, with an antiporter, there is no problem with Donnan equilibria: the system does not bung up. What is more, the passage of H^+^ down a natural gradient drives the efflux of Na^+^ ions. This has two beneficial effects, one immediate and the other longer term. The immediate benefit is improved coupling, assuming, as we do, that ancient ATPases were promiscuous for H^+^ and Na^+^, as modern ATPases of methanogens are [204]. Modern membranes are 2–3 orders of magnitude more permeable to H^+^ than to Na^+^, and there is no reason to suppose that early membranes would have been any different, especially in vents, where the presence of small organic acids uncouples H^+^ gradients but not Na^+^ gradients. So an antiporter transduces a natural proton gradient into a biochemical sodium gradient, which immediately improves coupling at essentially no energetic cost. Na^+^ extrusion is powered by the free geochemical proton gradient, not by active pumping (which consumes an energy budget that could otherwise be spent on carbon assimilation). Any improvement in coupling, of course, requires that the ancient ATPase could use Na^+^ ions, similar to how the modern one can [204,205], perhaps because hydronium ions (H_3_O^+^) have an identical charge and similar ionic radius to Na^+^ (ionic radii for unhydrated H_3_O^+^ = 115 pm, and for Na^+^ = 117 pm). A number of apparently ancient bioenergetic proteins found in both methanogens and acetogens, notably the ATPase and Ech, are promiscuous for H^+^ and Na^+^, which might be a relic of early coupling.

The longer term benefit relates to optimizing enzyme function by ionic balance. Most modern cells have low intracellular Na^+^ relative to K^+^, unlike the oceans, where Na^+^ concentration is much higher, 470 mM in oceans versus approximately 10 mM in cells [5]. Some universal and ancient proteins, notably those involved in translation, are optimized to this ionic balance, raising the question of how, if life arose in submarine alkaline hydrothermal vents, did intracellular Na^+^ concentration become lowered to this optimum? An H^+^/Na^+^ antiporter would readily explain how, but as the secondary consequence of a more immediate bioenergetic pressure.

The Na^+^/H^+^ antiporter also helps to solve the larger problem of the origin of active pumping. In the presence of free geochemical proton gradients, protocells in vents can adapt to a sodium-motive force, all the while being powered by a proton-motive force. Eventually, cell membranes became tight to Na^+^, if not H^+^. So how did active pumping originate?

Here, an important point needs to be made, lest the reader accuse us of invoking preadaptation with regard to pumping. There is no advantage to active pumping at regions of the vent where gradients are sharp by means of effluent flux. In distal regions of the vent, however, where alkaline effluent flux is impaired, energy coupling, and hence proliferation would cease. Only such protocells that ‘learned’—via the standard workings of natural variation and selection—to generate their own gradients in the context of their existing repertoire of redox reactions, could proliferate in such margins. And only such protocells could make the final transition to the free-living lifestyle. So there is clear proliferation benefit for the invention, by chance mutation, of proteins that can pump, and that benefit exists at regions of the vent where flux is impaired. This is likely the initial selective advantage of pumping.

What are the primordial active ion pumps? A simple solution is suggested by the sole coupling site of acetogens that lack cytochromes, Rnf. Electron transfer from ferredoxin to NAD^+^ drives the extrusion of Na^+^ via Rnf [147]. The problem of generating reduced ferredoxin in protoacetogens via flavin-based electron bifurcation at the hydrogenase step had been solved previously for the purpose of reducing CO_2_. The circuit was nearly complete, only redox balance remained to be achieved. Reoxidizing the NADH generated at the hydrogenase and Rnf reactions so as to synthesize methyl groups closes the stoichiometric loop, yielding an H_2_/CO_2_ dependent, acetate excreting entity that, upon cellularization (bubbling off, one might say) was energetically free to leave the vents.

Methanogens found a different and completely unrelated route, while also making use of flavin-based electron bifurcation, suggesting independent origins of active pumping. In the case of methanogens, the Na^+^-motive force is generated by pumping at the highly exergonic methyl transfer step (*ΔG*°′ = *ca* –30 kJ mol^−1^) from methyl-H_4_MPT to CoM by the methyl transferase (Mtr) complex, the only coupling site in methanogens that lack cytochromes [145]. Reoxidation of CoB-SH with methyl-CoM to generate the heterodisulfide CoM-S-S-CoB and methane establishes a redox balanced Na^+^ pumping circuit. In the protomethanogen lineage, this Na^+^ gradient could also be tapped via Ech for net CO_2_ to proceed. Regardless of the exact details, it appears that the deep divergence of overall design in methanogen and acetogen bioenergetics, despite their distinct commonalities, occurred within the vents.

From rather similar starting points (but with membranes that had already diverged), both methanogens and acetogens solved the problem of active pumping using an Na^+^/H^+^ antiporter, both relied on chemiosmotic ATP synthesis, and both evolved similar mechanisms of flavin-based electron bifurcation, using a similar set of hydrogenase proteins. But divergent stem lineages solved the details of the problem in different ways. And in both, flavin-based electron bifurcation gave rise to a balanced redox circuit and Na^+^ gradient generation via a single coupling site. Other elements of the more familiar respiratory chain, notably quinones, the Q cycle, and cytochromes *b* and *c*, arose later. This interpretation is supported by the remarkable homology of several subunits of complex I to the proteins discussed here: FeFe trimeric hydrogenases, NiFe hydrogenases, ferredoxin-reducing subunits, Ech and Na^+^/H^+^ antiporters [202]. The only further subunits required for true complex I function were quinone and NADH-binding subunits. We discuss the origin of quinones and cytochromes in §13, but it seems likely that they did indeed arise after Ech and Na^+^/H^+^ antiporters. In this case, it is also noteworthy that complex I itself possesses some of the same Na^+^/H^+^ promiscuity [206] as Ech and the ATP synthase do. The fact that the proton-motive force is more universal today than the sodium-motive force probably derives from evolutionary interpolation of quinones—which are always coupled to proton-dependent redox reactions—on these earlier, necessarily promiscuous origins [205].

Thinking things through, one additional aspect comes to the fore that is not immediately related to energetics. Namely, if the transition to the free-living state was truly independent in the archaebacterial and eubacterial lineages, as we suggest, then prokarytic cell division well could be predicted to have arisen twice independently. A look at the comparative genomics of cell division in archeabacteria and eubacteria provides evidence for the ubiquity of the FtsZ-derived system in eubacteria, and widespread occurrence of FtsZ in archaebacteria, but in addition the occurrence of ESCRTIII-related cell division machineries in some archaebacterial lineages [207]. Thus, while one can say that the ESCRTIII-related route is specific to archaebacteria, it is not obviously present in their common ancestor, while FtsZ (which seems to be a main cell division protein in methanogens) probably was [207]. Assuming that cell division had to evolve before cells escaped to the free-living state, what might cell division within rigid inorganic confines have looked like?

The example of spore formation in the acetogen *Acetonema longum* [208] is possibly instructive. Sporulation is a specialized case of cell division where the daughter cell grows inside the mother cell: some members of the clostridia, such as *Epulopiscium*, use the cell within a cell strategy for normal cell division [209]. During that process in *Acetonema*, the inner membrane of the mother cell becomes the outer membrane of the spore (the daughter cell), which grows within the mother cell and which initially posseses two membranes, inner and outer, one of which is ultimately shed [208]. This general kind of cell division has, not unreasonably, been suggested as the ancestral source of the outer membrane in Gram-negative bacteria; it might also represent an ancestral state of cell division reflecting a growth process within physical confinement.

## 10. Not enough energy?

Recently, Nitschke & Russell [210, p. 482] have argued that the Wood–Ljungdahl pathway ‘appears inadequate to drive reductions, condensations and biosyntheses required of an emergent metabolic cooperative’. The gist of this argument is that the H_2_–CO_2_ couple does not present enough free energy to get life started (although methanogens and acetogens do just fine). Their inference is that high-potential electron acceptors such as NO or NO_3_^–^ with midpoint potentials near or exceeding O_2_ must have been involved at the very earliest stages of chemical evolution [210,211], and in the latest formulation entailing oxidant-driven methane oxidation as the first step towards the synthesis of organic molecules [212]. A severe problem with that view, though, is that it demands that existence of a 1 : 1 molar ratio of high-potential acceptors for every organically incorporated carbon atom at the onset of primordial biochemistry; that would shift the oxidation state of such an environment dramatically.

This is especially true when one recalls that at the onset of biochemistry, there was little or no specificity in catalysis, such that a vast molar excess of ‘non-biogenic’ (side product) reduced carbon compound was required for the synthesis of every ‘biogenic’ (biologically useful) one [43]. The consequence is that in the presence of abundant high-potential acceptors of the type that methane oxidation [212] would demand, organic synthesis would probably just ‘go up in smoke’ being pulled towards CO_2_. The reason is because amino acid synthesis and cell mass synthesis, while requiring little if any energy input or even being exergonic under strictly anoxic hydrothermal vent conditions [36], become extremely endergonic even under very mildly oxidizing conditions, such as microoxic conditions corresponding to only 1/1000th of present oxygen levels [35].

Specifically, the synthesis of cell mass requires 13-fold greater energy input (or even more if NO_3_^–^ is the nitrogen source) under microoxic than under anoxic conditions [35]. That meshes with the observations of Heijnen & van Dijken [213] that anaerobic chemolithoautotrophs such as methanogens require of the order of 30–40 kJ to synthesize a gram of cell mass, whereas aerobes require of the order of 80–170 kJ to synthesize a gram of cell mass [35]. Thus, the argument that high-potential electron acceptors for methane oxidation were needed to get organic synthesis started [212] has the problem that their presence would tend to preclude accumulation of reduced organic compounds such as amino acids [35], because the reducing conditions would be gone. One might counter that these microoxic conditions apply to the oceans, whereas the internal compartments of hydrothermal vents are highly reducing, gases such as NO are as likely to traverse hydrophobic walls as CO_2_, and certainly more easily than protons, and would therefore inevitably alter the oxidation state within the vent. Indeed, McCollom & Amend [35] point out that ‘the primary control on the energetic requirements for biomass synthesis is the oxidation state of the environment’ which is consistent with the observations of Amend & Shock [34], who pointed out that in environments of high or even moderate reducing potential, the autotrophic synthesis of amino acids requires little energy input, in contrast to oxidizing environments. Thermodynamic considerations would strongly favour a reducing environment for the synthesis of life's first building blocks over an environment in which the synthesis of reduced carbon compounds had to take place against the workings of strongly oxidizing agents. An additional complaint of Nitschke & Russell [210] that ‘acetate has defied chemical synthesis in aqueous solution directly from CO_2_ and H_2_ in the laboratory’ probably relates to electron bifurcation, whereby aqueous acetate synthesis from CO_2_ with Fe^±0^ is facile [62].

And when it comes to the topic of strong oxidants, there is a lot to be said for molybdenum. Mo is involved in many interesting reactions, among them CO_2_ reduction in the initial steps of the acetogen and methanogen methyl synthesis pathways (figure 2) and N_2_ reduction in nitrogenase, which was recently shown to possess a carbon atom with only Fe ligands in the centre of its FeS cluster [214]. Mo is also involved in some reactions where it was long thought that only a strong oxidant such as O_2_ could do the job. A recent example is the characterization of a molybdoenzyme from *Sterolibacterium denitrificans* that anaerobically hydroxylates a tertiary carbon atom in sterol [215], a reaction hitherto thought to require O_2_. Another example is the anaerobic hydroxylation of ethylbenzene by ethylbenzene dehydrogenase, where Mo(VI) performs the oxidative step in the reaction mechanism [216].

## 11. A tree of tips

One might ask how phylogenomics stacks up against these concepts. Groups trying to work out the ‘phylogeny’ of prokaryotes [217] have come up with various schemes to classify groups, mainly based on the identification of some core set of genes that are concatenated and used to create a phylogenetic tree, a procedure laden with problems [218]. While such phylogenomic studies are worthwhile undertakings, they come with the heftiest of caveats. This is because even with massive amounts of data and refined molecular phylogenetic methods, it is a challenge to get the orders of mammals [219] or the orders of flowering plants [220] satisfactorily sorted out, and those processes span ‘only’ about 200 Myr into evolutionary time. Much, much harder are the early eukaryotic groups, which go back about 1.5 billion years [221], or even more difficult, linking eukaryotes to prokaryotes [222–224]. With that in mind, dismal appear the prospects of getting branching patterns sorted out for prokaryotes, which have been around for 3.8 billion years or more [30,164], assuming that there are any real branches in that phylogeny to be recovered in the first place [225], and the goal probably pushes phylogenetics beyond its limits.

**Figure 6.**
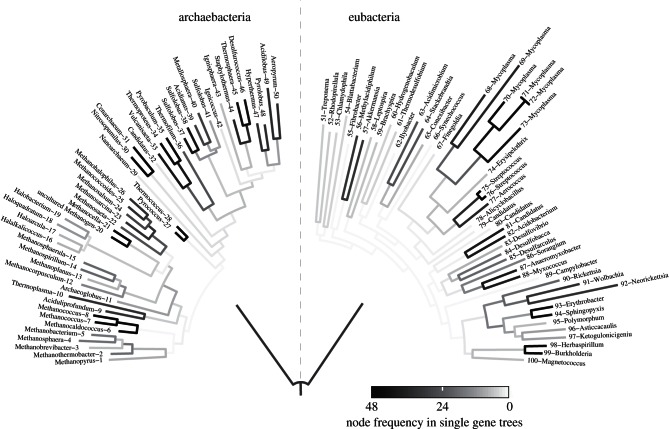
The ‘amazing disappearing tree’ of 48 universal genes for a 100 species set. A tree generated from a concatenated alignment of 48 universal genes, compared with its underlying single gene trees. The species sample comprises 50 archaebacteria and 50 eubacteria. To estimate the inconsistency between single gene trees and the concatenated tree, the frequency of each node in the concatenated tree was compared with its frequency within the single gene trees. The transparency of the branches reflects how often the associated node was present within the single gene trees. The 48 universal genes consist of the 31 genes that were previously identified as universal [226], and later used in phylogenetic analysis [217] namely (ArgRS, RNApol(a), LeuRS, metal-dependent protease, PheRS, GTPase, SecY, Rpl1, Rpl11, Rpl13, Rpl14, Rpl15, Rpl16/L10E, Rpl18, Rpl22, Rpl3, Rpl5, Rpl6, Rps11, Rps12, Rps13, Rps15/13E, Rps17, Rps2, Rps3, Rps4, Rps5, Rps7, Rps8, Rps9, Rps, SerRS), plus 17 additional genes (PRPP, AlaRS, PCNA homologue, RNApol(b), HisRS, Met-aminopeptidase, MetRS, PheRS beta subunit, ProRS, RecA, Rpl4, ThrRS, EfG, translation release factor, eIF5A, TyrRS, ValRS) that are present in this prokaryote sample, which contains no members with highly reduced genomes. The taxa were chosen for broad sampling. For this, proteomes of 1606 prokaryotes were retrieved from the RefSeq database (v03.2012) [166]. Pairwise sequence comparisons were run for the ribosomal protein L3. Based on these results, all prokaryotes were clustered by a hierarchical clustering algorithm. From each cluster 100 sample taxa (50 archaeabacteria and 50 eubacteria) were chosen. A complete list of genomes sampled is available in the electronic supplementary material.

What if we poll the genes that are used in such concatenation studies to see whether they individually tend to support the trees that they produce in concatenated analyses? We did that in figure 6. The result shows that the 48 core genes that can be distilled to be present in all of a small sample of 100 genomes recover, individually, the deep split between archaebacteria and eubacteria, and they recover recent phylogenetic signals at the tips of the tree (figure 6). The tree of life as currently constructed is based only on about 30 genes [217], which correspond to about 1 per cent of the genes found in an average prokaryotic genome; in that sense the tree of life is more the tree of 1 per cent [227]. But by viewing the tree in terms of how often different genes find the same branch for the concatenated tree they purport to uphold, what we see is not a tree of life, but a tree of tips [228]. In the deeper branches, not a single individual gene branches in the same way as the concatenated tree does. This underscores what Doolittle has been saying for some time [225,229], namely that scientists are unable to muster positive evidence that any given gene or set of genes has had the same history all the way back to the beginning of life's first cells.

Thus, while concatenated gene sets (analysed individually or in some concatenated manner) readily provide groups (a classification), the branching order of those groups remains obscure. But more importantly, even if one were, by some means, to get a branching pattern sorted (or agreed upon by consensus) for some core prokaryotic gene set, there remain incontrovertible and fundamental differences between classification of eukaryotic groups and classification of prokaryotic groups: the former is largely natural, but the latter is not [229]. For example, we can generate an approximate phylogeny for the evolution of vertebrates and map onto that phylogeny the origin of jaws, fins, lungs, feet, wings and so forth in a meaningful manner. But the same is not possible for prokaryotes because they readily spread their genes via lateral gene transfer (LGT), and what we find in modern genomes are useful collections of genes that represent viable strategies for survival in existing niches [225]. In other words, we cannot take any given prokaryote phylogeny and map out the linear evolution of physiological traits, because their evolutionary patterns are too complex, often marked by very sparse distributions.

Notwithstanding, it is possible to generate an argument, based on phylogenetic trees, that acetogens and methanogens are among the true latecomers in evolution, recently evolved and derived specialists [212]. But the branches in phylogenetic trees built from sequence data depend on many things, mainly things that go on inside computers [222]. And for each tree that suggests methanogens and acetogens to be latecomers [211], there is one to show that methanogens [222,224,230] and clostridia (acetogens) [217] branch at the base of their respective domains.

Why is the split between eubacteria and archaebacteria in figure 6 (and in many other such trees) so deep and so clear, while the next branches disappear? Figure 6 summarizes trees of universal genes. While they do not recover the middle ages of prokaryote evolution, they do tell us what we already know, that archaebacteria and eubacteria are very different. Under our current premises, their divergence corresponds to a comparatively short period of time, less than *ca* 400 Myr, that separates the advent of water at 4.2 Ga and evidence for life at 3.8 Ga [11,30]. Perhaps the simplest interpretation for this deep divergence is that it reflects the circumstance that the stem archaebacteria and stem eubacteria diverged at a time when proteins were still being invented and those that existed were getting better at what they do. In modern evolution, proteins evolve neutrally on the whole, functional constraints causing sequence conservation are the norm, and have been for most of Earth's history. But at the origin of genes, evolution was anything but neutral—it was fierce competition for organization of available matter into things that could organize matter best.

The examples encountered so far for independent origin of genes in archaebacteria and eubacteria clearly indicate that the invention of the full gene repertoire needed to exist as a free-living cell had not gone to completion in the confined universal ancestor. In the two stem prokaryote lineages, proteins were getting better at what they do (positive selection quickly fixes new mutations) and coming to rest in different regions of the fitness landscape on the plane of sequence space. This causes rapid sequence evolution, which translates into many accumulated sequence differences per unit time, which translates into long branches. Once proteins reached a certain level of optimization at what they do, functional constraints causing sequence conservation (slower mutation accumulation) became the norm.

## 12. What was present in the common ancestor?

We can look for proteins that are of sufficiently wide distribution to assume that they might have been present in the common ancestor of archaebacteria and eubacteria (table 1). If we opt for strict criteria as ‘present in all genomes’ then we would come up with a list of the roughly 31 proteins that various groups [217,226,232] have examined. An extended version of that list would also include many proteins that are less than 20 per cent identical in many pairwise comparisons. Our criteria for ‘widely distributed’ are more relaxed with respect to gene presence or absence but a bit more strict with respect to sequence identity. The protein families used by Nelson-Sathi *et al.* [231] to construct deep phylogenetic trees are convenient. The criterion of sharing at least 30 per cent amino acid identity was applied, because alignment and phylogeny procedures produce severe artefacts with amino acid sequences that share substantially less identity [233]. Concerning gene presence or absence, one can relax the stringency a bit, allowing for some loss, and ask whether a protein is present in at least one representative of various higher prokaryotic taxa corresponding to phylum, for example. In the study of Nelson-Sathi *et al.* [231], there were 11 higher archaebacterial taxa (75 genomes total) and 30 higher eubacterial taxa (1143 genomes total) sampled. Table 1 shows how many genes fulfil the distribution criteria for being present in (only!) at least one member each of 11/11 archaebacterial groups *and* in at least one member each of 20/30 higher prokaryotic taxa *and* sharing more than 30 per cent amino acid identity. Table 2 shows which genes those are, by annotation for the case of 106 genes that are present in at least one member each of 11/11 archaebacterial groups *and* in at least one member each of 20/30 higher prokaryotic taxa *and* that share more than 30 per cent amino acid identity.

**Table 1.** Genes generously universal across the great prokaryotic (archaebacterial-eubacterial) divide at varying taxonomic stringence. The 75 archaebacterial genomes fall into 11 major taxonomic groups, the 1143 eubacterial genomes fall into 30 major taxonomic groups, as given in detail in [231]. To be scored as present within a taxonomic group, the proteins are required to be approximately 30 per cent identical, which is stringent, but we generously allow for loss. Thus, a gene counted as present is present in at least 11/75 and 20/1143 genomes. Most numbers are much larger, of course. The annotations of those particular 106 gene families are listed by functional category in table 2.

	eubacterial						
	all 30	≥25	≥20	≥15	≥10	≥5	≥2
archaebacterial	—	—	—	—	—	—	—
all 11	54	93	**106**^a^	117	120	125	130
≥10	72	152	172	191	201	213	224
≥9	86	205	237	262	276	294	308
≥8	107	254	302	341	359	385	407

^a^There are 106 gene families present in at least *one member each* of 20 out of 30 major eubacterial taxonomic groups (roughly corresponding to NCBI taxonomy phyla) and present in at least *one member each* of all 11 major archaebacterial taxonomic groups sampled and roughly 30% identical in all comparisons.

**Table 2.** Functional annotation (using COG) of generously universal genes. List of 106 genes present in at least one member of all 11 archaeal groups and at least one member of 20 eubacterial groups (table 1).

no.	COG-id	category	product
I. information storage and processing			
1	COG0008	(J)	glutamyl- and glutaminyl-tRNA synthetases^a^
2	COG0012	(J)	predicted GTPase, probable translation factor^a^
3	COG0016	(J)	phenylalanyl-tRNA synthetase alpha subunit^a^
4	COG0017	(J)	aspartyl/asparaginyl-tRNA synthetases^a^
5	COG0024	(J)	methionine aminopeptidase^a^
6	COG0030	(J)	dimethyladenosine transferase (rRNA methylation)^a^
7	COG0048	(J)	ribosomal protein S12^a^
8	COG0051	(J)	ribosomal protein S10^a^
9	COG0060	(J)	isoleucyl-tRNA synthetase^a^
10	COG0080	(J)	ribosomal protein L11^a^
11	COG0081	(J)	ribosomal protein L1^a^
12	COG0086	(K)	DNA-directed RNA polymerase, beta’ subunit/160 kD subunit^a^
13	COG0090	(J)	ribosomal protein L2^a^
14	COG0092	(J)	ribosomal protein S3^a^
15	COG0093	(J)	ribosomal protein L14^a^
16	COG0094	(J)	ribosomal protein L5^a^
17	COG0099	(J)	ribosomal protein S13^a^
18	COG0100	(J)	ribosomal protein S11^a^
19	COG0124	(J)	histidyl-tRNA synthetase^a^
20	COG0185	(J)	ribosomal protein S19^a^
21	COG0442	(J)	prolyl-tRNA synthetase^a^
22	COG0480	(J)	translation elongation factors (GTPases)^a^
23	COG0525	(J)	valyl-tRNA synthetase^a^
24	COG0532	(J)	translation initiation factor 2 (IF-2; GTPase)^a^
25	COG0621	(J)	2-methylthioadenine synthetase^a^
26	COG5256	(J)	translation elongation factor EF-1 alpha (GTPase)^a^
27	COG5257	(J)	translation initiation factor 2, gamma subunit (eIF-2 gamma; GTPase)^a^
28	COG0049	(J)	ribosomal protein S7
29	COG0013	(J)	alanyl-tRNA synthetase
30	COG0072	(J)	phenylalanyl-tRNA synthetase beta subunit
31	COG0087	(J)	ribosomal protein L3
32	COG0089	(J)	ribosomal protein L23
33	COG0096	(J)	ribosomal protein S8
34	COG0097	(J)	ribosomal protein L6P/L9E
35	COG0098	(J)	ribosomal protein S5
36	COG0102	(J)	ribosomal protein L13
37	COG0103	(J)	ribosomal protein S9
38	COG0130	(J)	pseudouridine synthase
39	COG0162	(J)	tyrosyl-tRNA synthetase
40	COG0164	(L)	ribonuclease HII
41	COG0180	(J)	tryptophanyl-tRNA synthetase
42	COG0343	(J)	queuine/archaeosine tRNA-ribosyltransferase
43	COG0522	(J)	ribosomal protein S4 and related proteins
44	COG1093	(J)	translation initiation factor 2, alpha subunit (eIF-2 alpha)
45	COG1514	(J)	2′-5’ RNA ligase
46	COG2511	(J)	archaeal Glu-tRNAGln amidotransferase subunit E (contains GAD domain)
47	COG2890	(J)	methylase of polypeptide chain release factors
II. cellular process and signalling			
1	COG0396	(O)	ABC-type transport system involved in Fe-S cluster assembly, ATPase component^a^
2	COG0459	(O)	chaperonin GroEL (HSP60 family)^a^
3	COG0464	(O)	ATPases of the AAA+ class^a^
4	COG0489	(D)	ATPases involved in chromosome partitioning^a^
5	COG0492	(O)	thioredoxin reductase^a^
6	COG0533	(O)	metal-dependent proteases with possible chaperone activity^a^
7	COG0037	(D)	predicted ATPase of the PP-loop superfamily implicated in cell cycle control
8	COG0541	(U)	signal recognition particle GTPase
9	COG1180	(O)	pyruvate-formate lyase-activating enzyme
III. metabolism			
1	COG0020	(I)	undecaprenyl pyrophosphate synthase^a^
2	COG0078	(E)	ornithine carbamoyltransferase^a^
3	COG0082	(E)	chorismate synthase^a^
4	COG0112	(E)	glycine/serine hydroxymethyltransferase^a^
5	COG0126	(G)	3-phosphoglycerate kinase^a^
6	COG0127	(F)	xanthosine triphosphate pyrophosphatase^a^
7	COG0136	(E)	aspartate-semialdehyde dehydrogenase^a^
8	COG0142	(H)	geranylgeranyl pyrophosphate synthase^a^
9	COG0148	(G)	enolase^a^
10	COG0169	(E)	shikimate 5-dehydrogenase^a^
11	COG0171	(H)	NAD synthase^a^
12	COG0461	(F)	orotate phosphoribosyltransferase^a^
13	COG0498	(E)	threonine synthase^a^
14	COG0504	(F)	CTP synthase (UTP-ammonia lyase)^a^
15	COG0519	(F)	GMP synthase, PP-ATPase domain/subunit^a^
16	COG0540	[F)	aspartate carbamoyltransferase, catalytic chain^a^
17	COG1109	(G)	phosphomannomutase^a^
18	COG1155	(C)	archaeal/vacuolar-type H+-ATPase subunit A^a^
19	COG1156	(C)	archaeal/vacuolar-type H+-ATPase subunit B^a^
20	COG0002	(E)	acetylglutamate semialdehyde dehydrogenase
21	COG0005	(F)	purine nucleoside phosphorylase
22	COG0028	(EH)	thiamine pyrophosphate-requiring enzymes (acetolactate synthase, pyruvate dehydrogenase (cytochrome), glyoxylate carboligase, phosphonopyruvate decarboxylase)
23	COG0059	(EH)	ketol-acid reductoisomerase
24	COG0065	(E)	3-isopropylmalate dehydratase large subunit
25	COG0066	(E)	3-isopropylmalate dehydratase small subunit
26	COG0105	(F)	nucleoside diphosphate kinase
27	COG0119	(E)	isopropylmalate/homocitrate/citramalate synthases
28	COG0125	(F)	thymidylate kinase
29	COG0129	(EG)	dihydroxyacid dehydratase/phosphogluconate dehydratase
30	COG0137	(E)	argininosuccinate synthase
31	COG0160	(E)	4-aminobutyrate aminotransferase and related aminotransferases
32	COG0174	(E)	glutamine synthetase
33	COG0179	(Q)	2-keto-4-pentenoate hydratase/2-oxohepta-3-ene-1,7-dioic acid hydratase (catechol pathway)
34	COG0252	(EJ)	l-asparaginase/archaeal Glu-tRNAGln amidotransferase subunit D
35	COG0460	(E)	homoserine dehydrogenase
36	COG0462	(FE)	phosphoribosylpyrophosphate synthetase
37	COG0473	(CE)	isocitrate/isopropylmalate dehydrogenase
38	COG0499	(H)	S-adenosylhomocysteine hydrolase
39	COG0528	(F)	uridylate kinase
40	COG1013	(C)	pyruvate:ferredoxin oxidoreductase and related 2-oxoacid:ferredoxin oxidoreductases, beta subunit
41	COG1053	(C)	succinate dehydrogenase/fumarate reductase, flavoprotein subunit
42	COG1324	(P)	uncharacterized protein involved in tolerance to divalent cations
IV. unknown			
1	COG1163	(R)	predicted GTPase^a^
2	COG1245	(R]	predicted ATPase, RNase L inhibitor (RLI) homologue^a^
3	COG1782	(R)	predicted metal-dependent RNase, consists of a metallo-beta-lactamase domain and an RNA-binding KH domain
4	COG1690	(S)	uncharacterized conserved protein

^a^Present in at least one member of all 11 archaeal groups and at least one member of all 30 eubacterial groups.

The list depicts what sorts of functions might have been present as genetically encoded functions in the common ancestor, allowing generously for loss and keeping in mind that we cannot readily tell how much LGT has contributed to those gene distributions. With those caveats, the list tends to reflect a last common ancestor that had ribosomes, the genetic code, bits and pieces of cofactor biosynthesis, bits and pieces of amino acid biosynthesis, bits and pieces of nucleotide biosynthesis, and a fully fledged ATPase that could convert an ion gradient into chemically accessible high-energy bonds. The paucity of obvious components that would generate an ion gradient is striking. The list in table 2 is remarkably consistent with the view that the genes listed arose in an environment (an alkaline hydrothermal vent), where geochemically produced proton gradients existed and only had to be tapped. The presence of several tRNA modifying enzymes in the list is also congruent with our arguments about modified bases.

## 13. Haem and menaquinone

**Figure 7.**
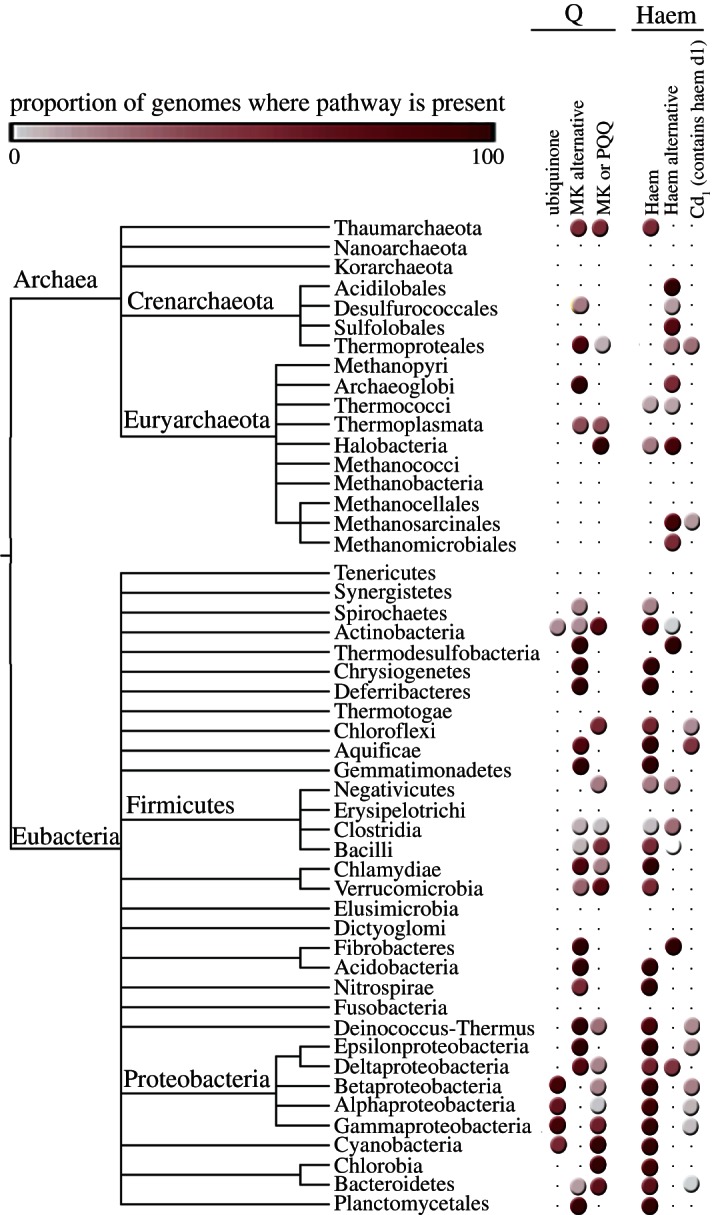
Distribution of quinone and haem biosynthetic pathways among 1606 prokaryotic genomes. The left part of the figure represents the organization of the selected taxonomic groups from the 1606 completed sequenced genomes (117 archaeal and 1489 eubacterial). The right part of the figure represents the proportion of genomes within a taxa where at least 70% of the genes involved in the pathway are present. Each column represents a different pathway. Homologous proteins involved in the several steps of ubiquinone (*ubiC*, *ubiA*, *ubiD/ubiX*, *ubiB*, *ubiH, ubiE*, *ubiF* and *ubiG*), menaquinone (MK) alternative (*MqnA*, *MqnB*, *MqnC* and *MqnD*) [235], menaquinone or phylloquinone (PQQ) (*MenF*, *MenD*, *MenH*, *MenC*, *MenE*, *MenB*, *MenA* and *UbiE*/*MenG*) [236], haem (*HemE*, *HemF*/*HemN*, *HemY*/*HemG* and *HemH*) and haem alternative (*AhbA*, *AhbB*, *AhbC* and *AhbD*) biosynthesis pathway were identified by BLAST [237]. The BLAST results were filtered for E values better than 10^−10^ and amino acid identities greater than or equal to 25 per cent. Owing to the high similarity between genes involved in haem d1 biosynthesis with genes from the haem alternative pathway [131], BLAST searches for the presence of cd1 nitrite reductase (the only enzyme containing haem d1) were also performed. In the genomes where both haem alternative pathway genes and cd1 nitrite reductase were present, the former were considered to be involved in haem d1 biosynthesis. Quinone biosynthesis distribution: ubiquinone is only present in the Eubacteria domain, mainly in proteobacteria (beta, alpha and gamma classes) and a few actinobacteria. It is an oxygen-dependent pathway being confined to aerobic organisms. The cyanobacterial ubiquinone hits reflect the presence of genes probably involved in plastoquinone biosynthesis instead. The two MK biosynthesis pathways are present in both prokaryotic domains although the MK alternative pathway has a broader distribution. The MK alternative pathway is the main pathway in both anaerobic and aerobic organisms such as archaeoglobi, thermoproteales, chrysiogenetes, deferribacteres, aquifecales, gemmatimonadetes, chlamydiae, fibrobacteres, acidobacteria, deinococcus-thermus, epsilonproteobacteria and deltaproteobacteria. The MK (and phylloquinone) ‘classical’ pathway is present in halobacteria, but it was acquired in their common ancestor by lateral gene transfer [231]. The classical pathway is also present in actinobacteria, gammaproteobacteria, cyanobacteria (PQQ and MK), chlorobia and bacteroidetes. Sulfolobales have benzothiophene quinone derivatives instead of typical quinones. Haem biosynthesis distribution: with very few exceptions (five out of 117 archaeal organisms surveyed here), the classical haem pathway is only present in the eubacteria domain. On the contrary, the alternative haem pathway is mostly confined to archaeal haem containing taxa and a few mostly anaerobic eubacteria (thermodesulfobacteria, gemmatimonadetes, clostridia, fibrobacterales and deltaproteobacteria). Interestingly, in some methanomicrobiales (organisms that do not contain cytochromes) genes coding for enzymes involved in the alternative haem pathway are present (*Methanoculleus marisnigri* JR1, *Methanoplanus petrolearius* DSM11571 and *Methanosphaerula palustris*). The role of the genes in these organisms is not clear and they might be involved in F_430_ synthesis instead.

The simplicity of the acetogen and methanogen bioenergetic pathways, not involving quinones or cytochromes and being replete in FeS centres, as well as their occurrence in strictly anaerobic chemolithoautotrophs, tends to speak in favour of their antiquity. An alternative view [211] has it that menaquinone (MK)-based-dependent proton gradient generation is the ancestral state of membrane bioenergetics, with a good portion of that argument resting on the wide distribution of MK among archaebacterial and eubacterial cells, and the occurrence of several ion translocating enzymes of bioenergetic membranes in some archaebacterial and eubacterial lineages. There are several problems with that view, though. Three problems with the argument for the ‘antiquity based on ubiquity’ of MK is that (i) methanogens do not have it at all, except derived methanogens that have acquired the genes for MK biosynthesis from eubacteria [231], (ii) members of the sulfolobales and acidobales synthesize benzothiophenes (quinone derivatives), but have no known MK pathway [234], and (iii) the distribution of the MK biosynthesis pathways across archaebacteria is generally sparse (figure 7),  whereby tests of the two possibilities underlying that sparse distribution, differential loss versus lineage-specific acquisitions, have not been reported. Another problem with the view that quinone-cytochrome-based ion-pumping systems are ancestral is that haems are lacking in all methanogens except the evolutionarily derived group of the methanosarcinales and that the archaebacteria haem biosynthesis pathway is unrelated to and arose independently from the eubacterial pathway [130,131]. We have plotted the distribution of the two haem and the two MK biosynthetic pathways across groups in figure 7. Besides the exceptions mentioned earlier, an interesting observation is the absence of both MK pathways in some quinone-containing organisms as for instance thermotogae and tenericutes. This might suggest the existence of an additional MK biosynthetic pathway. Of course one could also argue that non-enzymatic haem synthesis, which does work [238], is the ancestral state used by the common ancestor. However, a more problematic aspect of the cytochromes first model is that, in that view, both methanogens and acetogens are seen as very late and highly derived evolutionary lineages [212], whereas isotope evidence has it that methanogens are ancient [164] and comparative physiology would have it that the core biochemistry of both methanogens and acetogens is ancient [44,73,160].

If our premise is correct that acetogens and methanogens that lack cytochromes and quinones are the most ancient kinds of cells, and that the primordial organisms did not have basically everything and could not do basically everything in the bioenergetic sense, a question arises, namely who invented cytochromes and respiratory chains? Some acetogens have cytochromes, but at least those of *Moorella thermoacetica* [239] are lateral acquisitions, probably from eubacterial sulfate reducers, as sequence comparisons readily reveal. Some methanogens also have cytochromes but they are also lateral acquisitions [231]. The deltaproteobacterial sulfate reducers have regular cytochromes *b*, as well as multi-haem cytochromes *c* [172] synthesized via the alternative archaeal haem pathway. Sulfate reducers also have MK, but they also have some things in common with methanogens in terms of Hdr-related proteins. Sulfate reducers do not, however, have membrane integral cytochrome *bc* or cytochrome *b_6_f* complexes [172], and it is not yet clear how they use their MK pool, although a participation of electron confurcation, as discussed earlier, seems possible [197]. The circumstance that sulfate reduction is mainly a cytosolic process and that it likely involves flavin-based electron bifurcation (or confurcation) speaks for its antiquity. Indeed, given that there was surely no lack of sulfur substrates for sulfate reducers [179] at the vent–ocean interface, autotrophic sulfate reducers that use the acetyl-CoA pathway could, in principle, harbour a physiology that is nearly as ancient as that of acetogens and methanogens, requiring merely the additional evolutionary invention of cytochromes and quinones. The links between sulfur metabolism and methanogenesis point to the antiquity of both [163].

**Figure 8.**
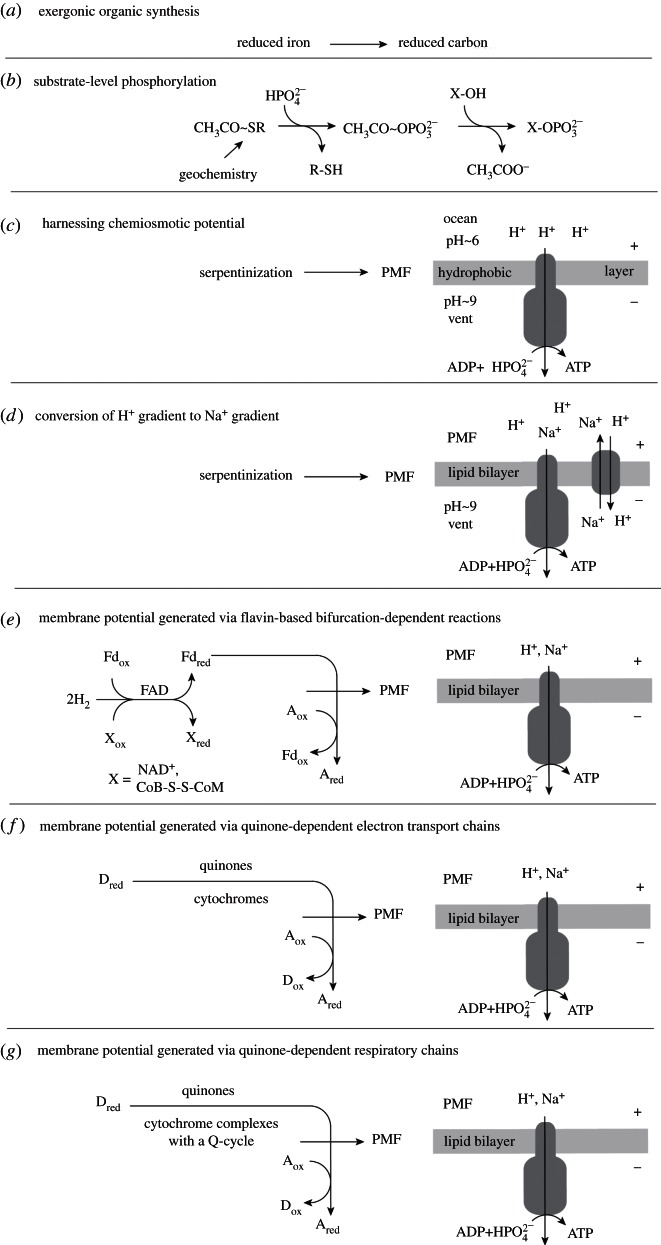
A summary diagram outlining a possible sequence of events in early bioenergetic evolution starting with (*a*) as the most ancient and ending with (*g*) as the most recent. Of course, respiratory chains are, generally speaking, ancient, just not as ancient as the bioenergetic processes in acetogens and methanogens that lack cytochromes or those in sulfate reducers, in our view (see text). The scheme in (*f*) could correspond to the situation in sulfate reducers; the scheme in (*g*) could correspond to the situation in *Paracoccus* [241] or *Rhodobacter* [242].

The prokaryotes who invented cytochrome *bc* type containing respiratory chains probably also invented the Q-cycle [240], the membrane phase and quinone-based analogue of flavin-based electron bifurcation, which is a cytosolic process so far. Among prokaryotes, flavins are far more universal than quinones. This suggests a possible sequence of events in the early evolution of energy conservation: (i) thioester-dependent substrate-level phosphorylations, (ii) chemiosmotic harnessing and the universality of ATP as energy currency, (iii) harnessing of Na^+^ gradients generated by H^+^/Na^+^ antiporters, (iv) flavin-based bifurcation-dependent ion gradient generation, (v) quinone-based (and, eventually, Q-cycle containing) proton gradient generation involving membrane integral cytochrome complexes and bona fide respiratory chains. All of these processes ultimately depend, even today, upon CO_2_ reduction with low-potential Fd_red_ (generated either chemosynthetically or photosynthetically), placing a reaction of the type ‘reduced iron → reduced carbon’ at the beginning of bioenergetic evolution, as outlined in figure 8.  The evolutionary advent of quinones affected chemiosmotic pumping efficiency in at least two ways: (i) via quinone-dependent electron bifurcation in the Q cycle, both in cytochrome *bc*_1_ and in cytochrome *b_6_f* complexes [243], and (ii) in complex I. The recent structure of complex I reveals how redox-dependent quinone movement introduces conformational changes across several adjacent antiporter subunits, causing them to pump in concert [244]. This generates a greater H^+^/2e^–^ stoichiometry than the hydrogenase-related precursors of complex I which, despite a similar subunit composition [202], pump without the help of quinones.

## 14. Conclusion

Here, we have specified some links between the energy releasing chemistry of a Hadean alkaline hydrothermal vent and the energy metabolism of particular groups of modern microbes. Energetic aspects of the origin of life are less widely discussed than RNA-oriented genetic aspects, and one can approach the problem either from a very general perspective, without linking early energetic models to modern bioenergetic configurations, or from a more specific perspective that aims to forge tangible connections between ancient chemical environments and modern microbial physiology. The latter approach requires stating some premises about what is ancient in modern biochemistry, and the further one delves into the physiology of acetogens and methanogens that lack cytochromes, the more ancient they look. Moreover, the more closely we compare their chemistry to processes at alkaline hydrothermal vents, the stronger the similarities become, and in terms of their antiquity the sulfate reducers lag not far behind. The circumstances that the acetogens and methanogens share the Wood–Ljungdahl pathway, the most ancient of six CO_2_ fixation pathways known [73], that they have cytochrome- and quinone-lacking forms (the only such groups among chemiosmotic prokaryotes known), that they are replete with FeS proteins [50,60]) as seen in figure 5, that they live in habitats hardly different from those on the early Earth [11,30], that their energy metabolism is centred around flavin-based electron bifurcation [143]—the soluble precursor of the Q-cycle—and that the energetic pillar of their energy metabolism is low-potential-reduced ferredoxin, are all best interpreted in our view as evidence reflecting their ancient and primordial nature. From the standpoint of energetics, it appears possible that life could have evolved from gases (H_2_, CO_2_, CO, N_2_, H_2_S, SO_2_) that reacted, with the help of transition metals, at the solid catalyst phase to produce aqueous organic compounds. Current views on the changes in free energy of biological systems, which are necessarily negative [14,29,39], as well as changes in entropy, which are close to zero [245,246], are compatible with that view. The antiquity of anaerobic chemolithoautotrophs seems as evident today as it did 40 years ago [44]. The ubiquity of chemiosmotic coupling as the ultimate source of net energy conservation [151] throughout the microbial world has, by comparison, come as a surprise. In the search for life's start, it is that peculiar energetic attribute of living things—the strict dependence upon chemiosmotic coupling—that makes alkaline hydrothermal vents [9] special. They go a long way towards closing the gap between acetogens, methanogens and the elements on early Earth.
